# A prediction for sepsis in adult patients with severe cerebrovascular disease from neurological intensive care unit

**DOI:** 10.3389/fneur.2025.1680602

**Published:** 2026-07-07

**Authors:** Haiyang Sun, Shuyun Sun, Yan Huang, Jingbo Sun, Chuanchuan Yu, Lixin Wang, Xiao Cheng

**Affiliations:** 1 Chinese Medicine Guangdong Laboratory /State Key Laboratory of Traditional Chinese Medicine Syndrome, The Second Affiliated Hospital of Guangzhou University of Chinese Medicine, Guangzhou, Guangdong, China; 2 Guangdong Provincial Key Laboratory of Research on Emergency in TCM, Guangzhou, Guangdong, China; 3 Affiliated Hospital of Shandong University of Traditional Chinese Medicine, Jinan, Shandong, China; 4 The First Clinical College of Shandong University of Traditional Chinese Medicine, Jinan, Shandong, China; 5 Affiliated Hospital of Qingdao Binhai College, Qingdao, China; 6 Department of Medical Statistics, School of Public Health, Sun Yat-sen University, Guangzhou, China

**Keywords:** acute moderate to severe stroke, sepsis, biomarker, IL-10, predictive model

## Abstract

**Background:**

Sepsis is one of the common causes of death in the neurological intensive care unit (NICU) stroke patients, the aim of this study was to evaluate the diagnostic performance of blood biomarkers studied for the early diagnosis of sepsis in ICU hospitalized patients with acute moderate to severe stroke, and to establish a that is specifically used to predict the occurrence of sepsis or not after stroke.

**Methods:**

A prediction model was built including 157 patients with severe cerebrovascular disease [including acute ischemic stroke (AIS) or cerebral hemorrhage (ICH)] who had National Institute of Health stroke scale (NIHSS) >14 or Glasgow coma scale (GCS) <8 from January 2020 to November 2022 in NICU. Laboratory parameters and clinical characteristics of the patients were collected as well as Enzyme-Linked Immunosorbent Assay (ELISA) to detect blood biomarkers IL-10, MIP-1β, TNF-α, nNOS, iNOS, MMP-9, S-100β, and ET-1 within 48 h after symptom onset. Multi-factorial logistic regression was used to construct for predicting sepsis in patients with acute moderate-to-severe stroke, and internal validation was evaluated using bootstrap validation. The performance of the graph was assessed based on its calibration, discrimination, and clinical utility.

**Results:**

The prevalence of sepsis in acute moderate-to-severe stroke patients was 12.1%. The GCS scores of patients with comorbidity sepsis were all lower than those of patients without sepsis, and the NIHSS scores were higher than those of patients without sepsis. Logistic stepwise regression was performed to identify 4 variables Hyperlipidaemia (*P* < 0.001), IL-10 (*P* < 0.001), NIHSS (*P* = 0.015), and Blood creatinine (*P* < 0.001), and to establish a prediction model for sepsis in acute moderate-to-severe stroke patients. The area under the curve (AUC) of the prediction model was 0.816 (95% CI: 0.721 ~ 0.911), and the calibration curve was well fitted, which has good clinical application value.

## 1 Introduction

Stroke is the second leading cause of death and the third leading cause of death and disability globally and its prevalence is still increasing (1). Infection as a risk factor for stroke affects approximately 30% of patients (2). Post-stroke infections are among the most significant complications that are considered as a determining factor for the stroke prognosis (3, 4). In patients who suffer from moderate-to-severe stroke, infections could lead to sepsis, adversely impacting their survival and recovery, and ultimately resulting in inferior clinical outcomes (5). Early and accurate identification of patients with sepsis with high mortality risk is critical for appropriate management of these patients. Although many clinical biomarkers have been investigated (6), few are currently applied in clinical practice because of the complexity and heterogeneity of sepsis. Therefore, the identification of useful biomarkers is imperative to provide timely and adequate interventions to patients with sepsis.

After stroke, the brain experiences acute-phase brain swelling and damage to the neurons, leading to the production of a large amount of inflammatory cytokines (7). ICU patients with severe ischemic stroke are more likely to develop sepsis during hospitalization due to a persistent inflammatory response (8). In a previous research study, it was found that there was a greater occurrence of pulmonary infections in patients who suffered stroke and were being treated in an intensive care unit (ICU). These patients were also more prone to developing sepsis. This increase in infections and sepsis led to a significant rise in the mortality rate during the hospital stay, as well as an increase in neurological deficits at 3 months after stroke. The study also found a correlation between higher NIHSS scores on admission, lower GCS scores, and the occurrence of pulmonary infections and sepsis during the hospitalization period (9). Therefore, significant work is needed to explore and verify the correlation between early blood biomarkers and the occurrence of sepsis in patients with severe cerebrovascular disease, and to identify the optimal combinations of biomarkers that can augment diagnosis, treatment, and good patient outcomes.

The main feature of sepsis is immune imbalance, and the two major immunological features in the development of sepsis are cytokine storm and immunosuppression. The highly heterogeneous nature of sepsis determines that no single biomarker can be used as a marker for its early diagnosis alone, and exploring the rational combinations of different cytokines with the existing indexes, especially anti-inflammatory factors and pro-inflammatory factors in such a combination that responds to the whole pathologic process, can achieve a rapid and accurate prediction of sepsis (10, 11). In this study, we detected blood biomarkers IL-10, MIP-1β, TNF-α, nNOS, iNOS, MMP-9, S-100β, and ET-1 within 48 h after symptom onset and combined them with other laboratory indexes, so as to established a prediction model for sepsis in patients with acute severe stroke in the NICU.

## 2 Materials and methods

### 2.1 Study population

**Figure 1:**
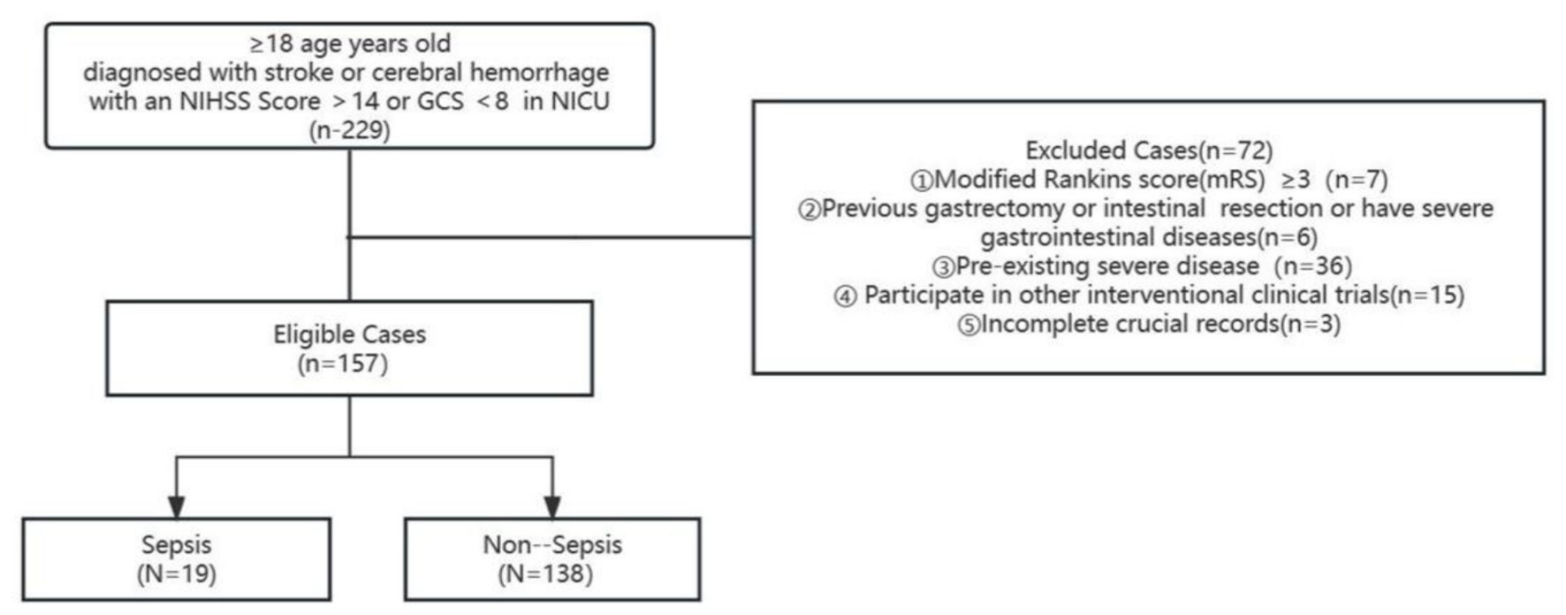
Flowchart of study participants.

In this study, prospective cohort study method was used to select patients with acute moderate or severe stroke who were admitted to the NICU of Guangdong Provincial Hospital of Traditional Chinese Medicine from January 2020 to November 2022 within 48 h. The study was conducted in accordance with the Declaration of Helsinki, and approved by Ethics Committee of Guangdong Provincial Hospital of Chinese Medicine (ZE2019-247-01, approved 5 December 2019). The study population was included in the study: ① Age > 18 years. ② AIS or ICH diagnosed by computed tomography (CT) or magnetic resonance imaging (MRI). ③ NIHSS > 14 or GCS < 8. The diagnostic criteria of sepsis were referred to: 2016 American Society of Intensive Care Medicine (ASICM) and European Society of Intensive Care Medicine (ESICM) joint publication of the definition and diagnostic criteria of sepsis 3.0 (12). (1) Suspected or confirmed infection with sequential organ failure assessment (SOFA) ≥ 2 points; (2) sepsis combined with circulatory failure, serum lactic acid (LAC) level > 2 mmol/L; (3) patients need to add vasoactive drugs to maintain Mean arterial pressure ≥ 65 mmHg. Exclusion criteria: (1) Modified Rankin Scale (mRS) >3; (2) patients with previous gastric or intestinal resection or severe gastrointestinal diseases; (3) patients with previous severe diseases, such as (1) advanced cancer; (2) severe pulmonary dysfunction FEV1 < 50%; (3) severe cardiac dysfunction EF% ≤ 30%; (4) severe hepatic failure. Child-Pugh score B-C; (5) severe renal failure GFR ≤ 30 ml/min or Blood creatinine ≥ 4 mg/dL. (4) Patients concurrently participating in other interventional clinical trials. (5) Patients treated with NSAIDs or corticosteroids. The study was approved by the local ethics committee and Flowchart is shown in Figure 1.

### 2.2 Statistical methods

After obtaining the clinical data, Continuous variables with normal distribution were expressed as mean with standard deviation (SD), non-normally distributed variables were expressed as median with interquartile range (IQR), and categorical variables were expressed as frequency and percentage (*n*, %). Normality of the distributions was assessed using graphs and the Shapiro-Wilk test. Depending on the distribution of continuous variables, continuous variables were compared between groups using the unpaired *t-*test or Wilcoxon rank sum test. Comparisons between binary categorical variables were made using the chi-square test or, in the case of small expected frequencies, the Fisher exact test. Two-by-two Spearman correlations were performed between continuous variables to assess correlation, Collinearity diagnostics were used to exclude high correlations between independent variables and multiple interpolation was used for missing values.

We introduced the internal verification method into the analysis. The least absolute shrinkage and selection operator (LASSO) method was used to reduce the risk of overfitting and reduce the dimensionality of the results by using sepsis prevalence as the dependent variable, and the variables with *P* < 0.05 in the results of the univariate logistic regression analysis were used to select the largest predictive characteristics of the risk factors and screen the predictors. The prediction model was established by multivariate logistic stepwise regression analysis. The modal plots of the prediction model were constructed, and the model was validated with 1,000 bootstrap resamples Bootstrap (1,000 repetitions) was used to validate the model internally, and the calibration curves were plotted to evaluate the calibration degree of the model. Binary logistic regression was used to analyze the subjects' work characteristics (ROC) curves to determine the discriminative ability of IL-10, NIHSS, and Blood creatinine as prognostic predictors. The discriminative validity was further evaluated by analyzing the ROC curves and calculating the AUC, sensitivity, and specificity. The clinical decision curve (DCA) was also constructed to evaluate the clinical application value of the model and quantify the net benefit within the threshold probability range. The results show that the model has good stability because of its consistent performance under different verification methods.

For all analyses, *P* < 0.05 was considered statistically significant. Statistical analysis was performed using IBM SPSS 26.0, Graph Pad Prism 9.0, and R version 4.2.0.

## 3 Results

### 3.1 Baseline characteristics of patients

As present in Table 1, 157 patients were included in the final analyses, of whom 12.1% had sepsis, 65.61% had hypertension, 27.39% had type 2 diabetes mellitus, 21.02% had coronary artery disease, and 8.92% had chronic kidney disease. In these individuals, stroke severity was moderate to severe with a median GCS score of 10, and a median NIHSS score of 19.

**Table 1:** Baseline characteristics of patients.

Variable	Total	Non-Sepsis	Sepsis	*T/χ^2^*	*P-value*
	N=157	N=138	N=19		
Female, *n* (%)	91 (57.96%)	81 (58.70%)	10 (52.63%)	0.252	0.616
Age, years	66.48 ± 13.77	65.72 ± 13.96	72.00 ± 11.09	3.527	0.062
Surgery, *n* (%)	80 (50.96%)	75 (54.35%)	5 (26.32%)	5.251	0.022
Height, m	1.65 [1.58;1.70]	1.65 [1.60;1.70]	1.65 [1.55;1.70]	0.915	0.340
Weight, kg	65.00 [55.00;70.00]	65.00 [56.00;70.00]	62.50 [50.00;74.50]	0.168	0.682
BMI, Kg/m^2^	23.88 ± 3.90	23.87 ± 3.64	23.92 ± 5.54	0.003	0.958
Hyperlipidaemia, *n* (%)	38 (24.20%)	29 (21.01%)	9 (47.37%)	Fisher	0.02
Hypertension, *n* (%)	103 (65.61%)	91 (65.94%)	12 (63.16%)	0.057	0.811
DM, *n* (%)	43 (27.39%)	39 (28.26%)	4 (21.05%)	0.436	0.509
CHD, *n* (%)	33 (21.02%)	26 (18.84%)	7 (36.84%)	Fisher	0.128
CKD, *n* (%)	14 (8.92%)	13 (9.42%)	1 (5.26%)	Fisher	1
ET-1, pg/mL	201.22 [121.23;312.97]	213.13 [119.09;313.46]	142.49 [125.23;299.35]	0.174	0.678
IL_6, pg/mL	34.10 [16.60;77.10]	34.10 [19.01;86.32]	18.22 [12.45;68.67]	13.804	< 0.001
IL_10, pg/mL	778.74 [668.74;1,088.74]	777.81 [665.24;1,043.90]	861.13 [752.08;1,396.40]	10.583	0.001
iNOS, pg/mL	8.86 [7.88;10.41]	8.89 [7.90;10.40]	8.71 [7.52;10.12]	0.024	0.877
MIP_1β_CCL4, pg/mL	328.02 [292.56;371.16]	326.04 [290.41;370.91]	348.61 [309.26;398.01]	0.065	0.799
MMP_9, pg/mL	38.93 [30.08;55.91]	38.93 [30.48;55.42]	37.06 [29.78;122.57]	0.763	0.384
S_100_β, pg/mL	392.38 [270.73;452.85]	394.73 [270.05;450.36]	356.88 [322.68;466.05]	0.558	0.456
TNF_α, pg/mL	123.52 [80.44;157.85]	123.18 [81.62;151.94]	140.12 [74.73;169.83]	3.013	0.085
nNOS, pg/mL	8.29 [5.15;11.83]	8.46 [5.08;11.83]	7.79 [5.92;9.29]	0.01	0.922
GCS	10.00 [6.00;12.00]	10.00 [6.00;12.00]	6.00 [4.00;9.00]	7.246	0.008
NIHSS	19.00 [15.00;28.00]	18.00 [15.00;26.00]	33.00 [16.00;35.50]	9.026	0.003
NLR	7.59 [3.59;13.76]	7.06 [3.65;13.24]	11.68 [2.57;21.01]	2.043	0.155
LMR	2.17 [1.29;3.80]	2.25 [1.32;3.71]	1.53 [1.01;5.25]	2.026	0.157
PLR	173.57 [113.96;282.42]	172.86 [113.99;253.39]	273.17 [107.47;399.80]	0.862	0.355
PPR	23.30 [18.79;30.87]	23.72 [19.08;31.23]	22.14 [12.81;27.41]	3.625	0.059
NMLR	8.01 [3.94;14.84]	7.61 [3.99;13.83]	13.02 [2.75;22.58]	2.944	0.088
Neutrophil, 10^9^/L	8.84 [5.74;12.76]	8.75 [5.86;12.71]	9.58 [4.84;13.02]	0.001	0.969
LymphocyteCount, 10^9^/L	1.27 [0.85;1.94]	1.27 [0.88;1.93]	1.19 [0.56;2.46]	0.072	0.789
MonocyteCount, 10^9^/L	0.56 [0.43;0.73]	0.56 [0.44;0.74]	0.49 [0.42;0.72]	0.571	0.451
PlateletVolume, 10^9^/L	9.20 [8.60;10.00]	9.20 [8.60;10.00]	9.40 [9.00;11.20]	0.132	0.717
PlateletCount, 10^9^/L	218.00 [178.00;267.00]	218.00 [185.00;272.50]	206.00 [152.50;224.00]	1.158	0.284
BNP, pg/ml	87.30 [34.50;291.30]	85.15 [34.65;293.70]	87.30 [37.45;178.50]	0.347	0.557
CRP, mg/dL	22.94 [6.17;73.83]	21.66 [6.17;67.04]	43.50 [8.02;117.00]	3.712	0.056
PCT, ng/ml	0.16 [0.06;0.48]	0.16 [0.06;0.48]	0.15 [0.11;0.57]	0.001	0.980
OxygenationIndex, mmHg	280.90 [228.50;352.00]	277.00 [230.00;345.72]	300.00 [194.62;366.45]	0.129	0.720
BloodCreatinine, umol/l	80.00 [66.00;97.00]	81.50 [67.00;99.00]	71.00 [50.50;91.00]	3.261	0.073
UrineCreatinine, mmol/d	9,540.00 [6,948.00;12,097.00]	9,719.00 [7,107.50;12,218.75]	8,624.00 [6,462.00;10,557.00]	1.171	0.281
Albumin, g/L	40.30 [35.00;43.00]	40.45 [35.05;43.08]	39.00 [35.25;42.25]	0.58	0.448
Prealbumin, g/L	218.31 ± 70.51	221.53 ± 69.07	194.96 ± 78.26	2.392	0.124
Transferrin, mg/L	1.79 [1.54;2.07]	1.79 [1.54;2.07]	1.86 [1.40;2.09]	0.387	0.535
Hemorrhage Combined With Ischaemia, *n* (%)	33 (21.02%)	27 (19.57%)	6 (31.58%)	Fisher	0.237
Hydrocephalus, *n* (%)	11 (7.01%)	8 (5.80%)	3 (15.79%)	Fisher	0.132
Brainhernia, *n* (%)	31 (19.75%)	27 (19.57%)	4 (21.05%)	Fisher	1
Infectious_diseases, *n* (%)	112 (71.34%)	97 (70.29%)	15 (78.95%)	0.612	0.434
Hypoproteinaemia_Anemia, *n* (%)	48 (30.57%)	43 (31.16%)	5 (26.32%)	0.185	0.667

a Continuous variables with normal distributions are presented as means ± standard deviation (SD). b Continuous non-normally distributed variables are presented as medians (P25, P75). c Categorical variables are presented as *n* (%). SD, Standard deviation; IQR, interquartile range (expressed as 25th−75th percentile); BMI, Body Mass Index; IL-6, Interleukin-6; PCT, procalcitonin; NIHSS, National Institute of Health stroke scale; NLR, neutrophils/lymphocytes ratio monocyte; LMR, lymphocytes/monocytes ratio; PPR, platelet count/platelet volume ratio; IL-10, Interleukin-10; TNF-α, tumor necrosis factor-α; MIP-1β, macrophage inflammatory protein-1β; nNOS, neuronal nitric oxide synthase; iNOS, inducible nitric oxide synthase; MMP-9, matrix metalloproteinase-9; S-100β, CNS specific protein-100β; ET-1, endothelin-1.

### 3.2 Screening for predictive factors

In the training set, we first screened potential predictors of the outcome event (*P* < 0.1) using univariate logistic regression analysis. These variables include age, Surgery, Hyperglycaemia, IL_6, IL_10, GCS, NIHSS, PPR, NMLR, Blood Creatinine. For the identified variables, we further selected significant features (with non-zero coefficients) through the Least Absolute Shrinkage and Selection Operator (LASSO) logistic regression algorithm. The optimal parameter configuration was determined using 10-fold cross-validation, with coefficients identified by lambda values corresponding to one standard error (1 se) of the minimum distance deviation. Subsequently, we conducted multivariate logistic regression analysis (stepwise method, bidirectional) on these screened variables. As shown in Table 2. Multivariate logistic model analysis showed that 4 factors were independent predictors of sepsis in patients with acute moderate to severe stroke based on the following results (*P* < 0.05). Hyperlipidaemia OR = 3.806 (95%CI:0.991,15.019); IL_10: OR = 1.002 (95%CI: 1.000~1.003); NIHSS: OR = 1.091 (95% CI: 1.014~1.181); Blood Creatinine: OR = 0.967 (95%CI: 0.939~0.989).

**Table 2:** Parameters of multivariate logistic stepwise regression.

Variable	Estimate	SE	Statistic	OR(95%CI)	*p* Value
(Intercept)	−3.418	1.262	−2.708		0.007
Hyperlipidaemia	1.73	0.618	2.8	3.806 (0.991,15.019)	0.005
IL_10	0.001	0.001	1.995	1.002 (1.000,1.003)	0.046
NIHSS	0.084	0.036	2.345	1.091 (1.014,1.181)	0.019
BloodCreatinine	−0.032	0.012	−2.55	0.967 (0.939,0.989)	0.011

SE, standard error; IL-6, Interleukin-6; IL-10, Interleukin-10; NIHSS, National Institute of Health stroke scale.

### 3.3 Risk prediction development

**Figure 2:**
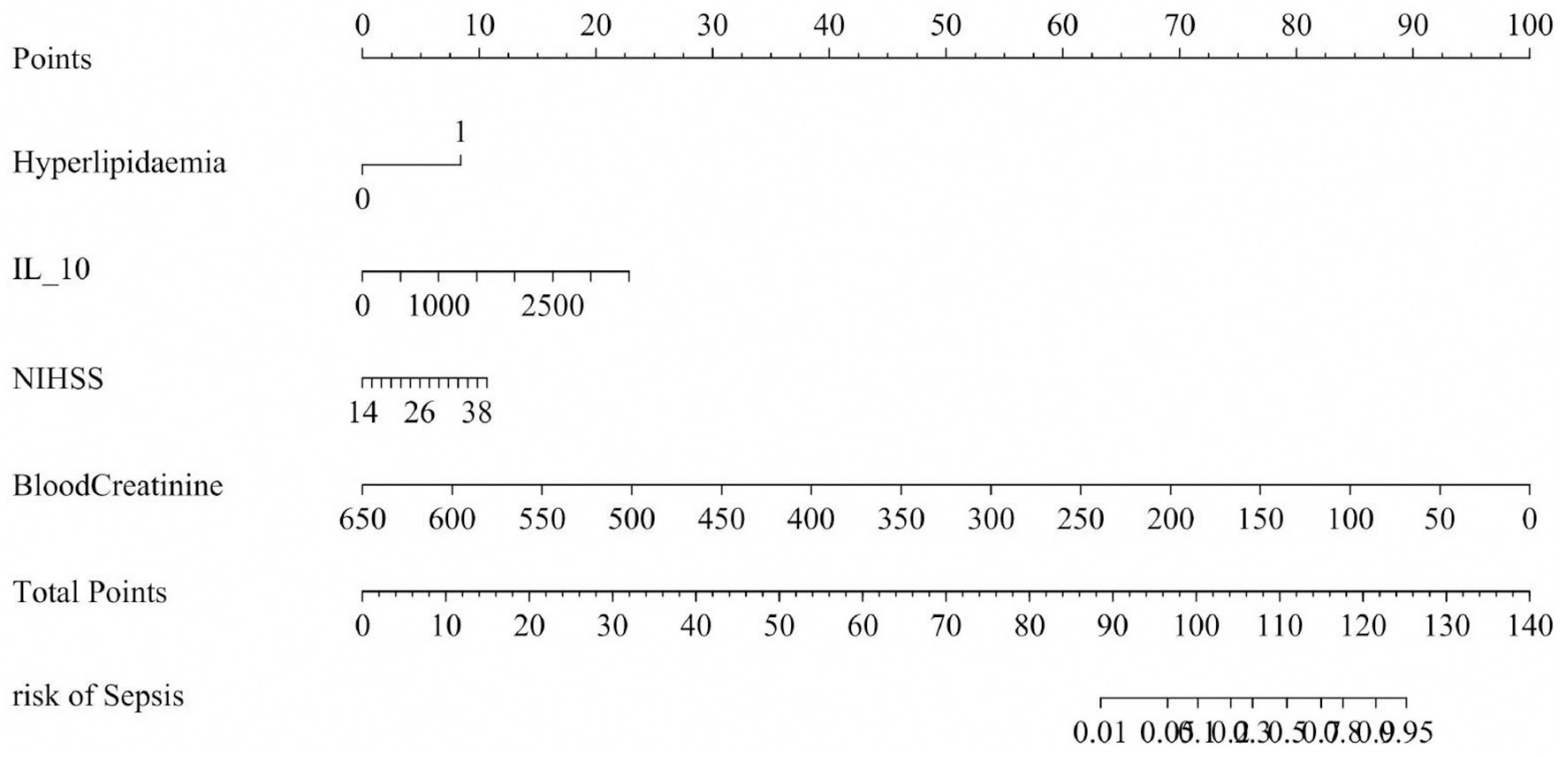
To estimate the probability of sepsis in adult patients with severe cerebrovascular disease from NICU.

The logistic regression model was constructed based on the above 4 factors (Table 2), after which these 4 factors from the logistic regression model were integrated to the (Figure 2). For each patient, higher total points indicated a higher risk of Sepsis.

A for sepsis was developed and integrated with the predictors. Find the predictor points on the uppermost point scale that correspond to each patient variable and add them up. The total points projected to the bottom scale indicate the probability of sepsis. IL-10 Interleukin-10, NIHSS National Institute of Health stroke scale. In the figure, the first row shows the score corresponding to a single variable. A vertical line is drawn above each variable to obtain the corresponding score. The total score is obtained by adding up the scores corresponding to each variable. Find the corresponding score in the last row (total score), then draw a vertical line downward to find the point in the last row, and the risk probability of the predicted outcome can be obtained.

### 3.4 Validation of the model

#### 3.4.1 Discrimination

**Figure 3:**
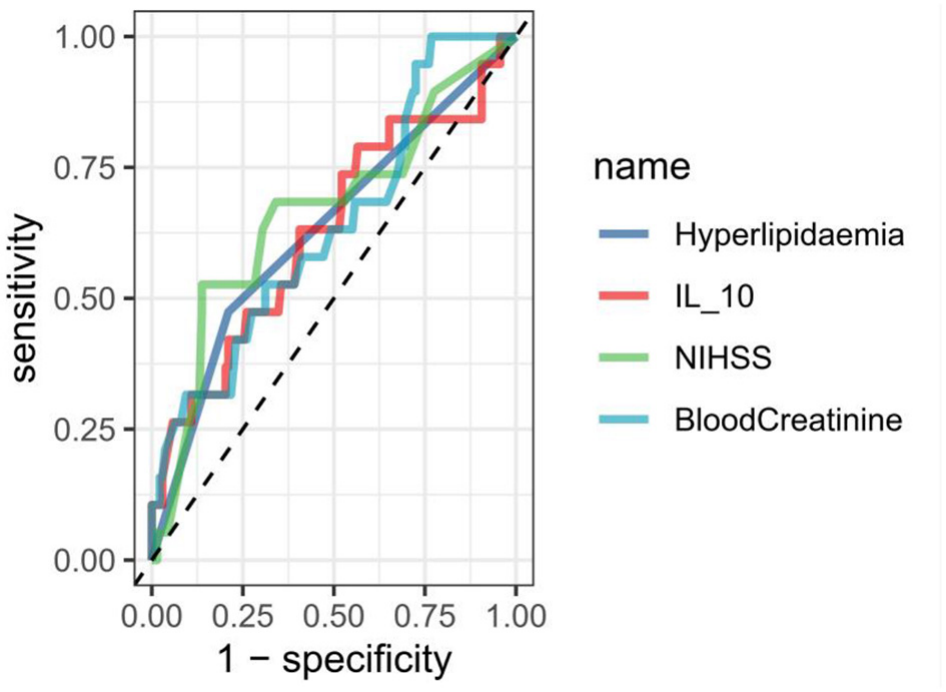
ROC curve evaluation was applied to determine the predictive power of the model for sepsis. ROC curve analysis showed that Hyperlipidaemia1 (AUC = 0.632, 95% CI: 0.511–0.752); IL-10 (AUC = 0.628,0.479–0.777), NIHSS score (AUC = 0.644,95% CI: 0.519–0.809), Blood Creatinine (AUC = 0.637,95% CI: 0.501–0.774), and the AUC value of the combined model was 0.816 (95% CI: 0.721–0.911).

AUC (area under the ROC curve) is the area under the ROC curve, which is used to measure the performance of a classifier. The closer the AUC value is to 1, the better the performance of the classifier, when 0.78 < AUC ≤ 0.83: it represents the strong efficacy of the model. The AUC is plotted in Figure 3. This model has excellent discriminative power with an AUC of 0.816 (95% CI: 0.721–0.911) in the dataset (Figure 3).

#### 3.4.2 Calibration

**Figure 4:**
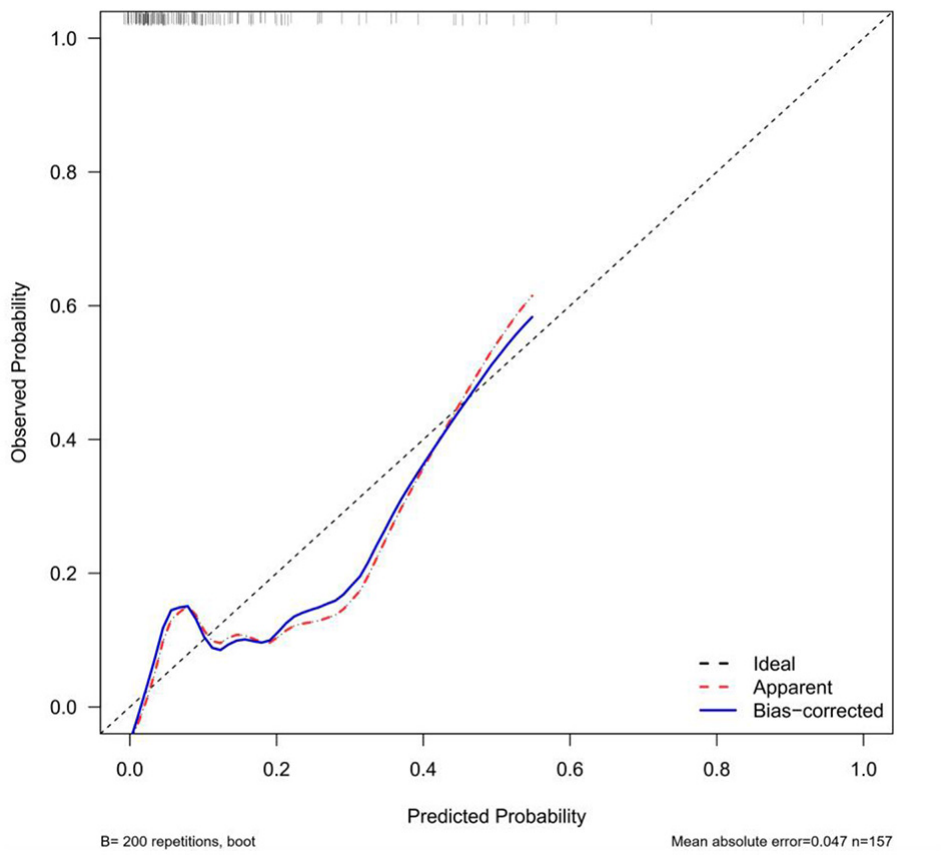
Calibration plot of the prediction model. The dashed black line represents perfect calibration. The red dashed line indicates the apparent calibration curve, and the blue solid line represents the bias-corrected curve based on 200 bootstrap resamples. The close alignment of the bias-corrected curve with the ideal line demonstrates good calibration performance. Mean absolute error = 0.047.

The calibration curve was plotted in Figure 4, which was evaluated with the bootstrap sampling validation test in the dataset. This model was well calibrated, with no significant difference between the predicted and the actual probability.

#### 3.4.3 Decision curve

**Figure 5:**
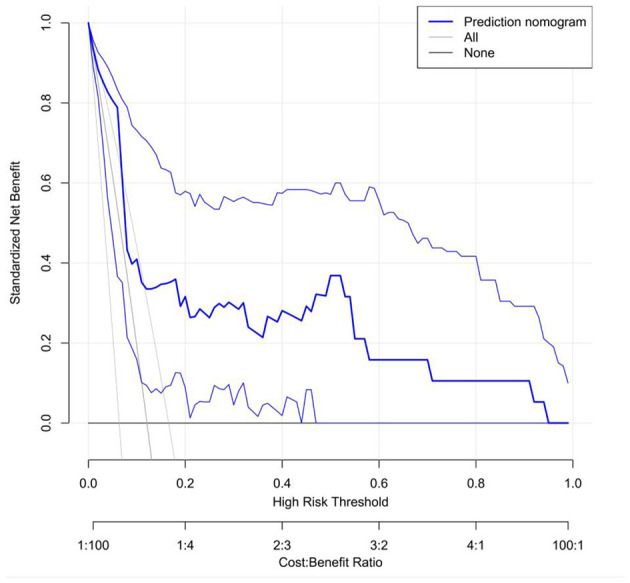
Decision curve analysis for the nomogram. The clinical application value of DCA in assessing the risk of sepsis. The y-axis represents the net benefit, and the x-axis represents the threshold probability. The blue line indicates the assumption that all cases possessed sepsis in hospital with all receiving the interventions. According to the decision curve, the threshold probability >20 for the patient would be more beneficial to use this graph to predict in-hospital sepsis risk.

Decision curve analysis is shown in Figure 5.

## 4 Discussion

Stroke is the second leading cause of death globally that predisposed to sepsis. Sepsis-related morbidity and mortality are major problems in NICU including relatively severe stroke patients. How to make early judgments on the occurrence of sepsis after stroke has important reference value for evaluating the prognosis of patients and guiding treatment. This study detected the differential changes in blood biomarker in the early stages of stroke onset and established a model for predicting the occurrence of in-hospital sepsis in patients with acute moderate-to-severe stroke in NICU combined with laboratory and clinical information. In this study, some infection-related data were not fully available during the early hospitalization period (particularly when biomarkers were sampled within 48 hours of admission), and event limitations constrained the number of variables included. Additionally, certain infection evidence was confirmed several days after birth, falling under the category of “outcome/mediator” rather than “baseline predictors.” Our study showed that advanced age was a risk factor for sepsis in stroke patients, and that sepsis patients had higher prevalence of hyperlipidemia, NIHSS score, IL-10 values, and intervention and craniotomy reduced the risk of sepsis development, while GCS score-IL-6 values were lower. Additionally, our study showed that Hyperlipidaemia,IL-10, NIHSS, and blood creatinine were predictors of the development of sepsis during hospitalization in acute moderate to severe stroke patients.

Hyperlipidemia is a common early complication of post-stroke septicemia and can further aggravate the functional impairment of other organs (13). As a part of the body's congenital immunity, septic hyperlipidemia can promote the occurrence of septicemia in patients with moderate and severe stroke, and is an important factor determining the prognosis of septic patients. Previous studies have shown that plasma TC, HDL-C, and LDL-C levels are independent risk factors for septic AKI, and the lower the blood lipid level, the higher the risk of death (14). A Mendelian randomization and population study focused on the causal relationship between metabolic markers and sepsis-related outcomes, providing for the first time evidence of causal indication through observational and genetic evidence, and showing that stroke, hyperlipidemia and sepsis intensive care unit admission were associated (15). In addition, as a traditional risk factor for stroke, hyperlipidemia may have a potential role in the neuroinflammatory response and brain-peripheral interference of sepsis and stroke (16).

As we know, the cyclical nature of the inflammatory response and multifactorial-induced immunosuppression are central features of sepsis (17–19). Evidence has been presented that pro- and anti-inflammatory cytokines are released shortly after the onset of sepsis (20, 21) and continue to be released in tandem throughout the course of the disease (22–24). Brain-immune crosstalk is a key player in the evolution of sepsis and stroke, both at its acute and recovery phase, influencing neurotoxic and neuroprotective mechanisms (25).^.^Early studies have shown that the expression of inflammatory cytokines occurs in the early stages of acute ischemic stroke (ACIS) before the emergence of an inflammatory cellular response, and that IL-10, a pleiotropic biokine with both immunosuppressive and immunostimulatory effects, which has a wide range of bioactivities, is an important anti-inflammatory cytokine associated with cerebral ischemic injury, the process of injury repair, and has neuroprotective and neuro trophic effects (26). In the early stage of ischemic injury, the more severe the injury, the higher the serum IL-10 level, the more dramatic the change in serum IL-10 concentration, the faster the ischemic stroke progresses, and the more neurological function is impaired, and IL-10 plays an important role in the pathophysiological process of ischemic stroke progression. While TNF-α and MMP-9 are associated with inflammatory responses, their levels may be influenced by acute brain injury itself and the time window for detection, resulting in insufficient predictive value demonstrated in this study. In contrast, IL-10, as an anti-inflammatory cytokine, better reflects the severity of immune imbalance and shows a more direct correlation with sepsis development. Therefore, IL-10 demonstrates stronger explanatory power both statistically and biologically. Two subsequent studies have shown that high IL-10 levels on admission or 6 h after ischemia are important predictors of brain injury and lung infection (27, 28)^.^ In patients with cerebral hemorrhage, studies have shown that higher plasma IL-10 levels on admission are associated with hematoma expansion and the development of complications (29). Surveys such as that conducted by Zhang Wei have shown that IL-10, IL-17, and PCT tests all have high diagnostic value in sepsis patients, that combinations of the three tests outperform individual tests in terms of diagnostic performance, and that the combination of the three tests has a higher overall clinical benefit rate (30)_._ The discrepancy may be due to confounding factors or interactions between variables. In the multivariable context, IL-10′s independent predictive role became more apparent when adjusted for other clinical indicators, which is a key strength of multivariable modeling. Our study showed that the combination of four tests, hyperlipidemia, IL-10, NIHSS, and blood creatinine, has a good diagnostic and predictive value in sepsis patients after moderate to severe stroke.

Sepsis is defined as life-threatening organ dysfunction and can be determined using the Sequential Organ Failure Assessment (SOFA) score. The applicability of the SOFA score is limited in patients not receiving intensive care treatment (31). The National Institutes of Health Stroke Scale (NIHSS) was initially used mainly for the evaluation of neurological status in stroke patients, and the scale is concise, highly reliable and accurate with commonality and low sensitivity (32)^.^ which is useful in the rational assessment of the condition, individualized treatment, prediction of the prognosis, and correct health education, and is widely used in neurosurgery, interventional radiology, neurology, and critical care Medicine. In a prospective pilot study, the baseline NIHSS score was essential for prediction of acute ischemic stroke outcomes, followed by age; whereas traditional comorbidity index contributed little to the overall model (33). A Retrospective cohort study of Medicare claims data by Amit Kumar indicate that NIHSS is significantly associated with 30-day mortality in Medicare patients with ischemic stroke and significantly improves discriminant property relative to the Elixhauser comorbidity index (34). Meanwhile, a Meta-analysis including 59 articles showed that the OR of NIHSS score in predicting post-stroke pneumonia was 1.07 (95% CI = 1.05–1.09), further emphasizing the use of NIHSS score in the development of post-stroke complications (35). Sepsis is one of the most serious complications in patients with severe stroke and is a recognized risk factor for death (36).

The third independent predictor of Sepsis in our study was blood creatinine. Elevated blood creatinine is one of the markers of acute kidney injury (AKI), AKI and sepsis carry consensus definitions (37)^.^ creatinine as a marker of renal dysfunction, which predisposes patients to infection and sepsis. An analysis of the MIMIC-III Clinical Database illustrated an inverted M-shaped curve between the blood urea nitrogen/creatinine ratio (BCR) and mortality in patients with infectious shock. BCR was identified as an easily accessible and independent prognostic biomarker in patients with infectious shock, and higher BCR was associated with an increased mortality in these patients (38). Evidence from basic research suggests that the role of local microcirculation and inflammatory signaling (including ischemia-reperfusion injury, oxidative stress, and renal tubular apoptosis) is more important in AKI mechanisms (39–41). Meanwhile, due to the crosstalk between the nervous system and the kidneys, in acute kidney injury (AKI), the damaged kidneys can have further adverse effects on the central nervous system, which can further affect the occurrence and prognosis of stroke complications (42). A meta analysis of 31 different studies that included 504,503 critically ill patients from a variety of ICUs and a separate random-effects meta-analysis showed that 13 different risk factors such as diabetes, hypertension, and baseline elevation of creatinine were associated with AKI with increasing age (43)^.^

The main strength of this study lies not in identifying a single strong biomarker, but in integrating clinical and laboratory predictors into a combined model, which significantly improved predictive performance. The value of this comprehensive model represents the central contribution of our work.

## 5 Conclusions

In conclusion, our study indicated that IL-10, NIHSS, blood creatinine acquired within 48 h after NICU admission were significant predictors of sepsis in moderate and severe stroke patients. Moreover, the model constructed with IL-10, NIHSS, and blood creatinine might have a robust predictive value, which is of great importance for clinicians to make personalized management for sepsis in severe stroke patients. This study provides valuable preliminary evidence for predicting post-stroke sepsis and establishes a foundation for future large-scale research.

## 6 Limitations

Furthermore, we noted that this study had some limitations. (1) Due to the limited sample size, given the relatively small number of sepsis events (*n* = 19) in this study, the event-per-variable (EPV) ratio in the final model was below the commonly recommended threshold of 10–15 events per predictor variable. Such a low EPV increases the likelihood of model overfitting, which may lead to optimistic performance estimates and reduced reproducibility in external populations (44). Although penalized regression (LASSO) and internal bootstrap validation were applied to minimize this bias, the results should still be interpreted with caution. Model stability is still limited, the findings should be considered exploratory and preliminary. (2) This was a single-center retrospective study, thus, a larger sample and multi-center cohort or prospective studies would be needed to verify. (3) This study mainly predict the occurrence of sepsis in patients with moderate to severe cerebrovascular disease, which limits its promotion and application in patients with mild stroke. (4) Our study lacked verification of external validity, the adaptive scope of the model in this study needed to be further verified. (5) Strokes in this study included ischemic and cerebral hemorrhage, although they share some pathophysiological mechanisms, have different etiologies, which will be further classified into subgroups in future studies. (6) Such as stroke lesion volume, type of occlusion in ischemic stroke, medications prior to the onset of the stroke, and the proportion of patients who were not considered to have been treated with intravenous thrombolysis and/or endovascular therapy in ischemic stroke may also be a limitation of this study. We are currently conducting a multicenter study with a larger cohort, which will allow for external validation and potential refinement of the model. As a key focus of future model improvement, we plan to systematically collect information on infection type, microbiological results and invasive procedures (central venous catheter, duration of mechanical ventilation, etc.) and immunosuppression status in future studies.

## Data Availability

The original contributions presented in the study are included in the article/Supplementary material, further inquiries can be directed to the corresponding authors.
