# Supplementary figures and images for: A prediction for sepsis in adult patients with severe cerebrovascular disease from neurological intensive care unit
Source: Front Neurol. 2026 Jul 7;16:1680602. doi: 10.3389/fneur.2025.1680602 (PMC13384880; doi:10.3389/fneur.2025.1680602)

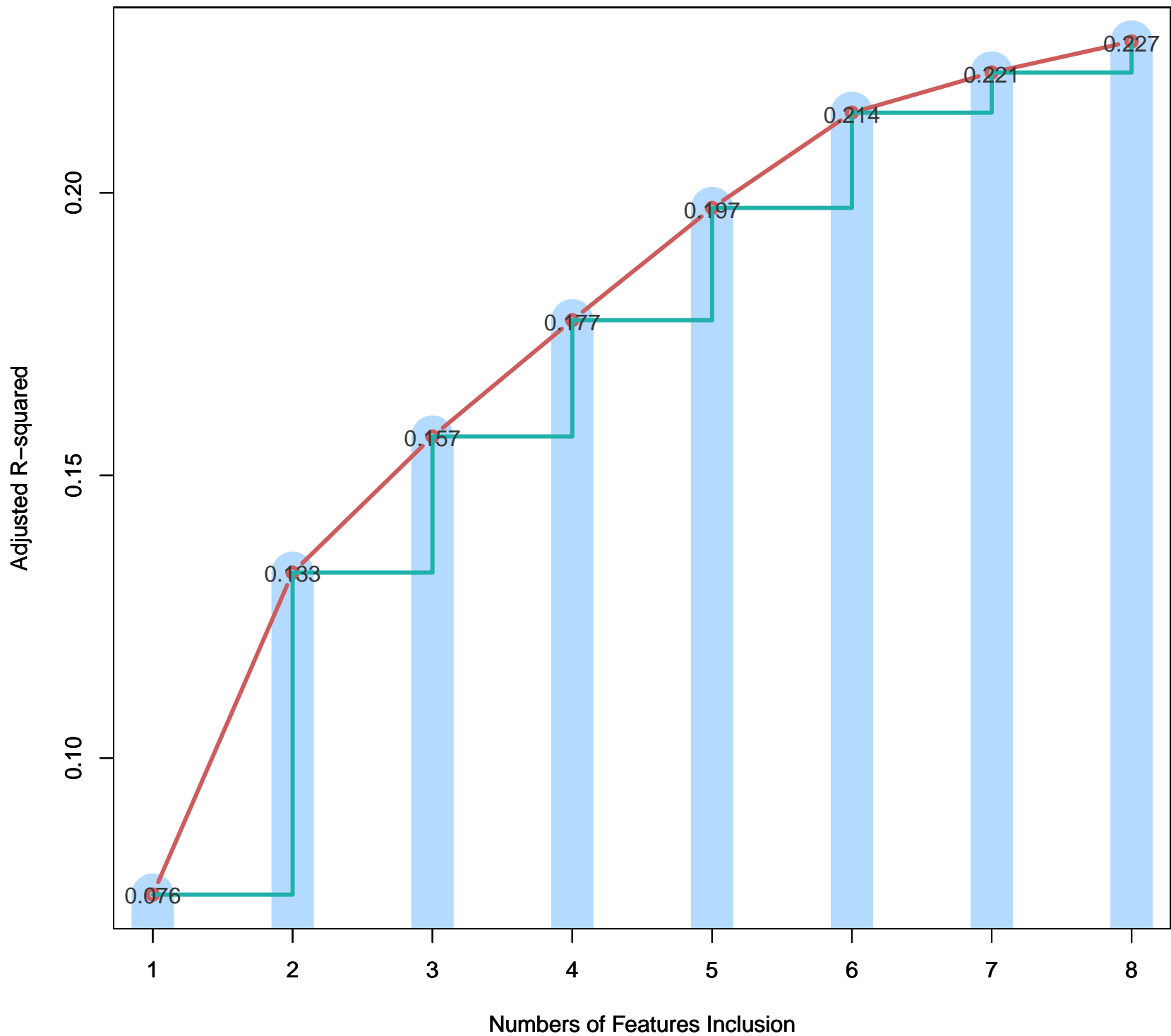

Supplement: Supplementary file 1 [file Data_Sheet_1.zip › 1.Adjusted.R-squared.pdf]

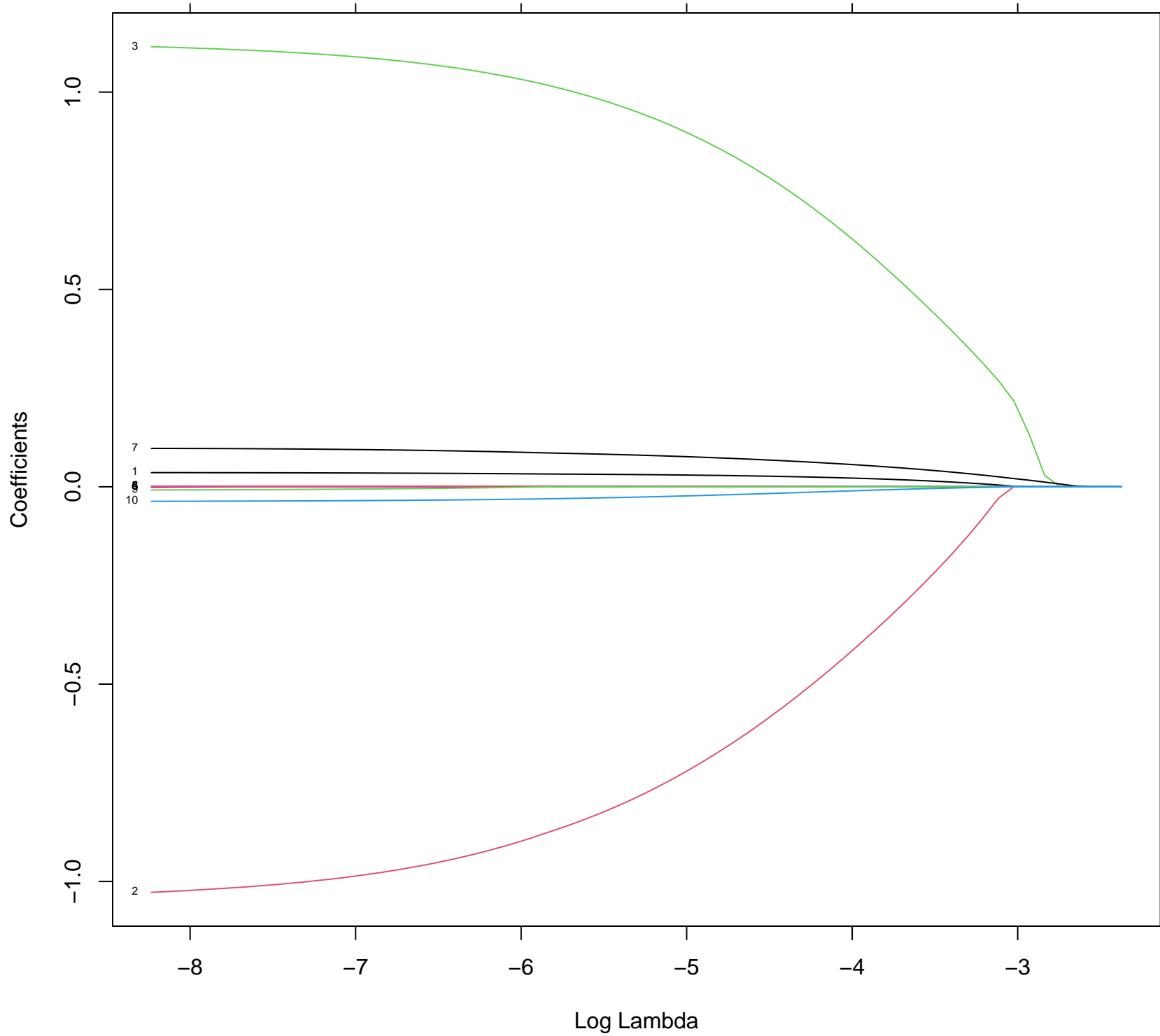

Supplement: Supplementary file 1 [file Data_Sheet_1.zip › 10.ridge.or.lasso.reg.pdf]

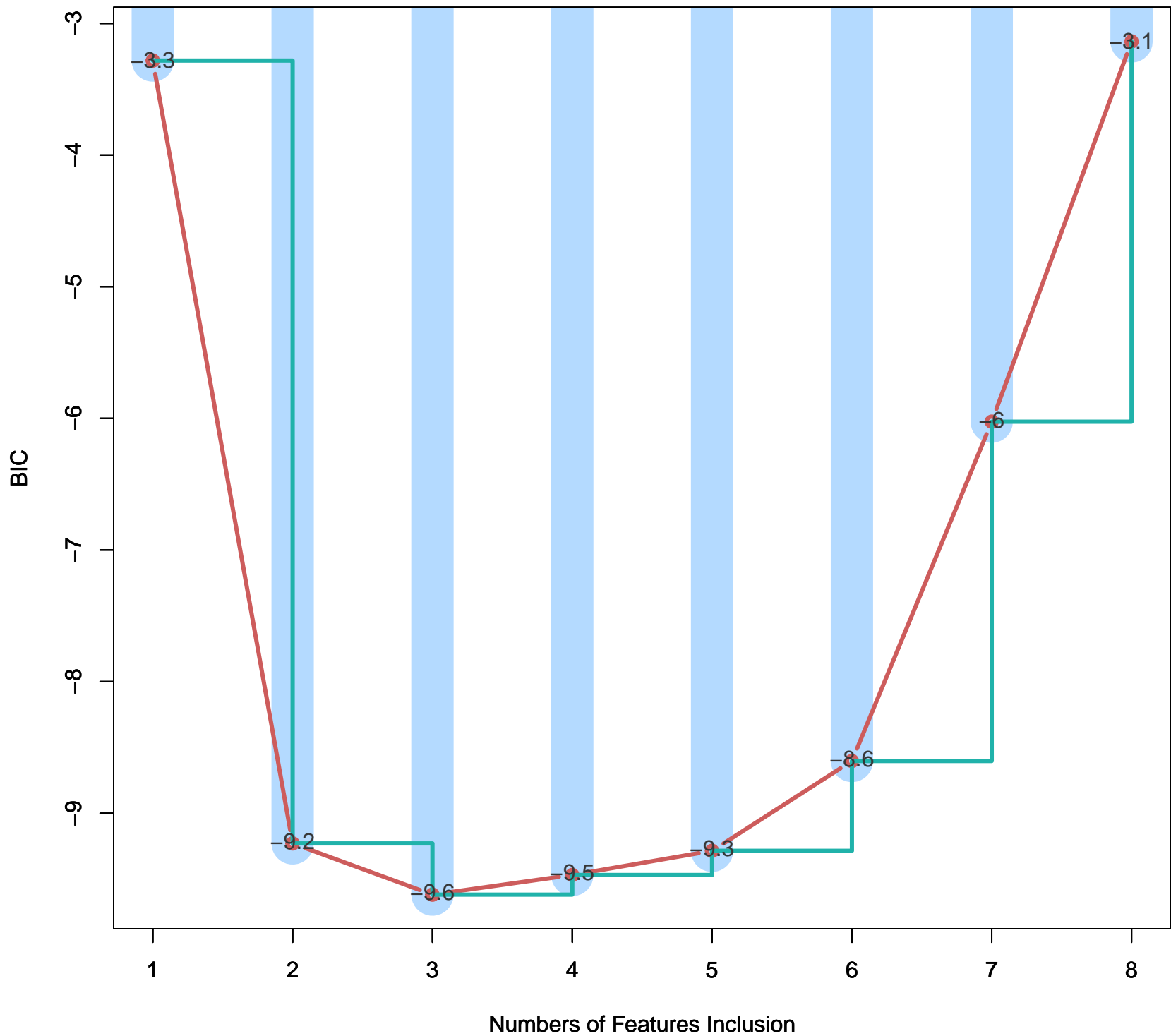

Supplement: Supplementary file 1 [file Data_Sheet_1.zip › 2.BIC.pdf]

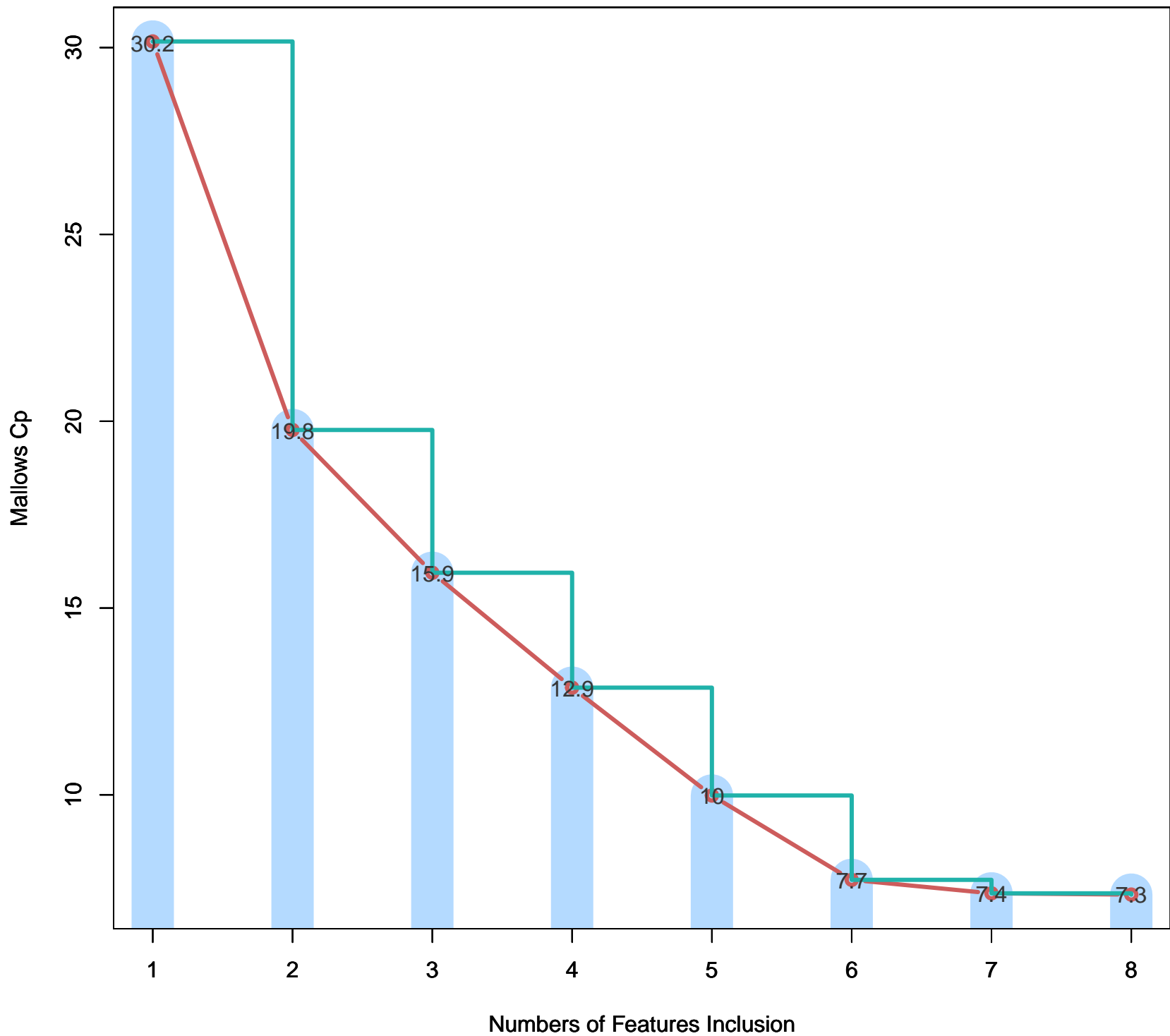

Supplement: Supplementary file 1 [file Data_Sheet_1.zip › 3.Mallows.Cp.pdf]

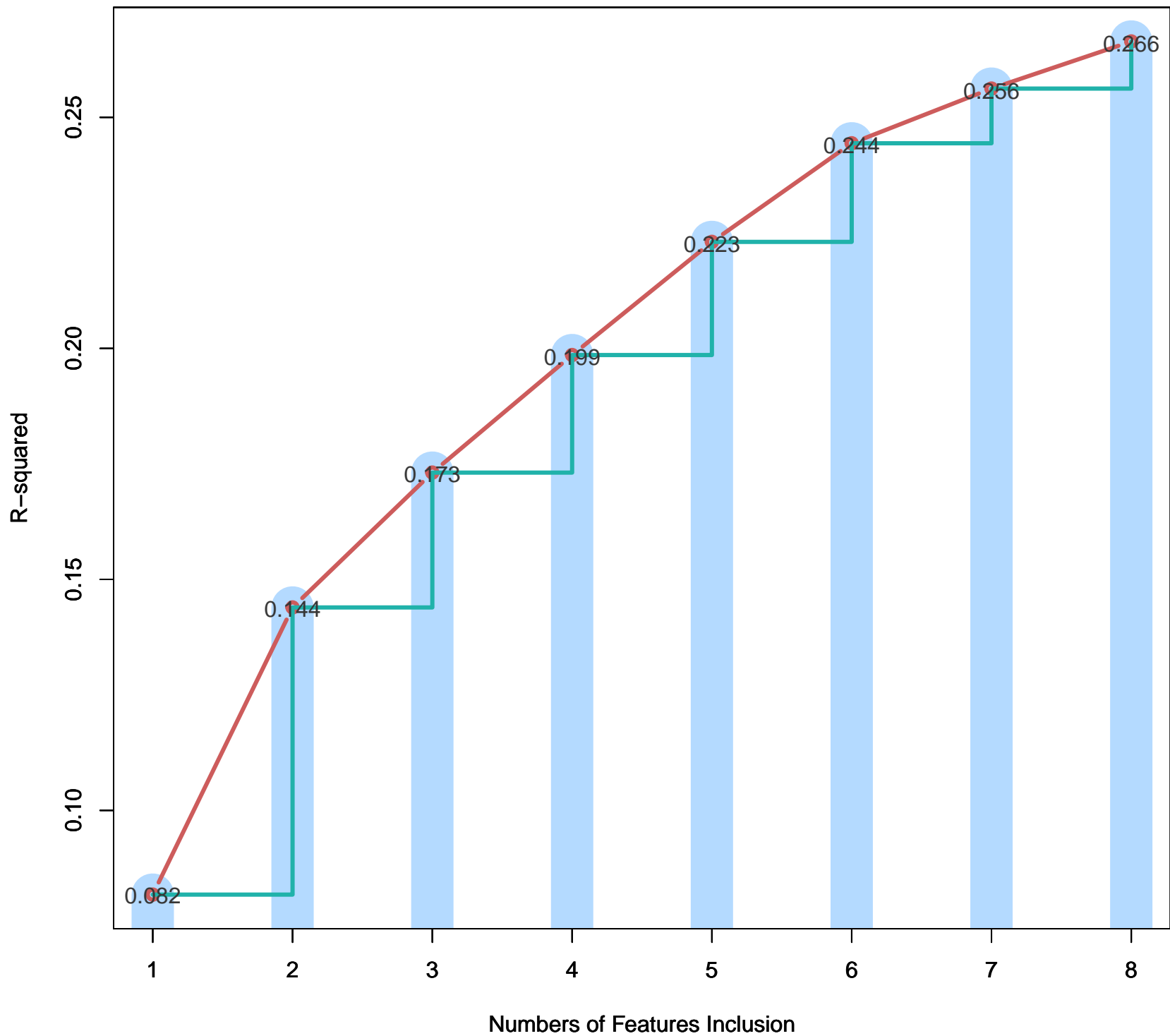

Supplement: Supplementary file 1 [file Data_Sheet_1.zip › 4.R-squared.pdf]

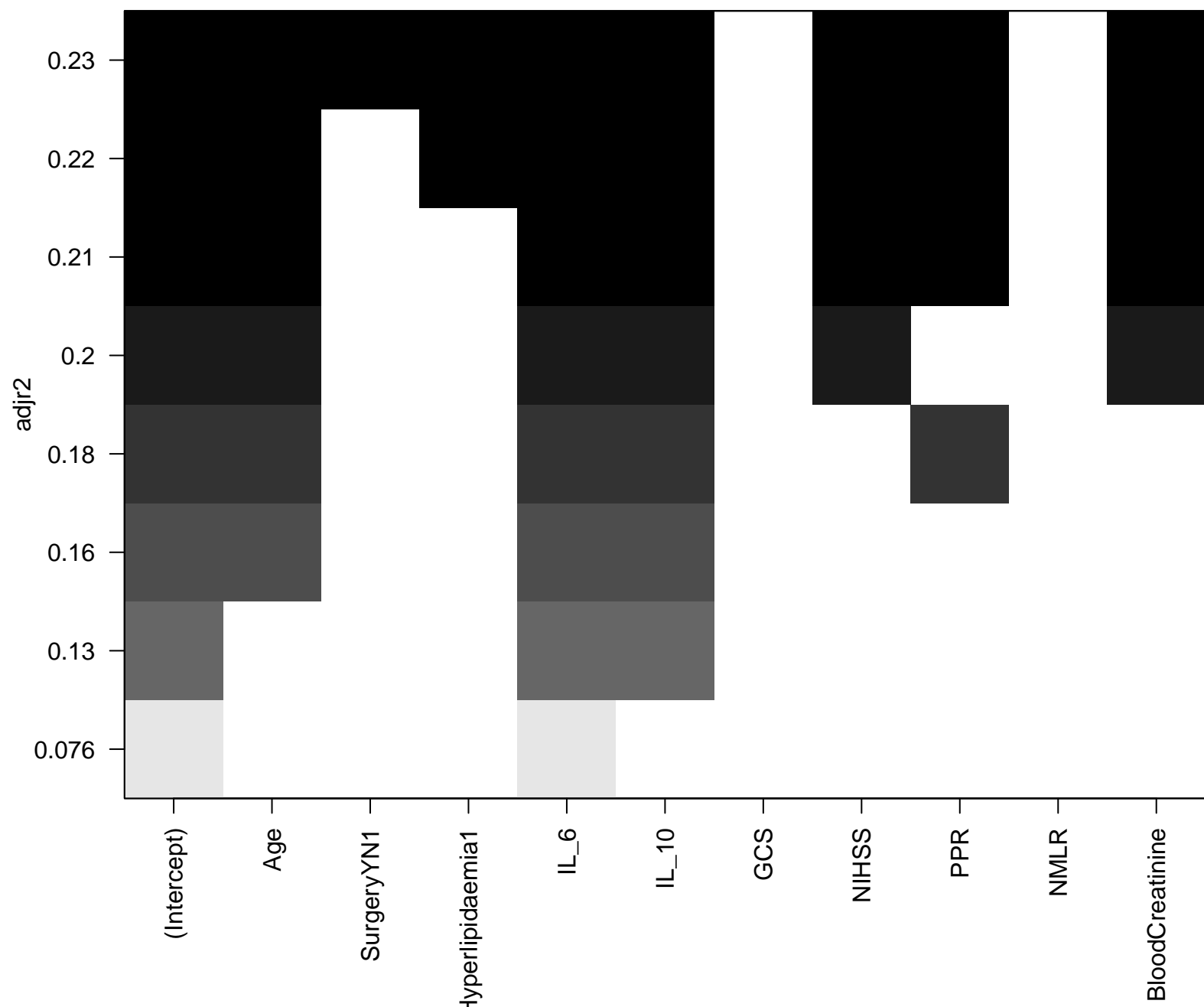

Supplement: Supplementary file 1 [file Data_Sheet_1.zip › 5.Adjusted.R-squared.BSR.pdf]

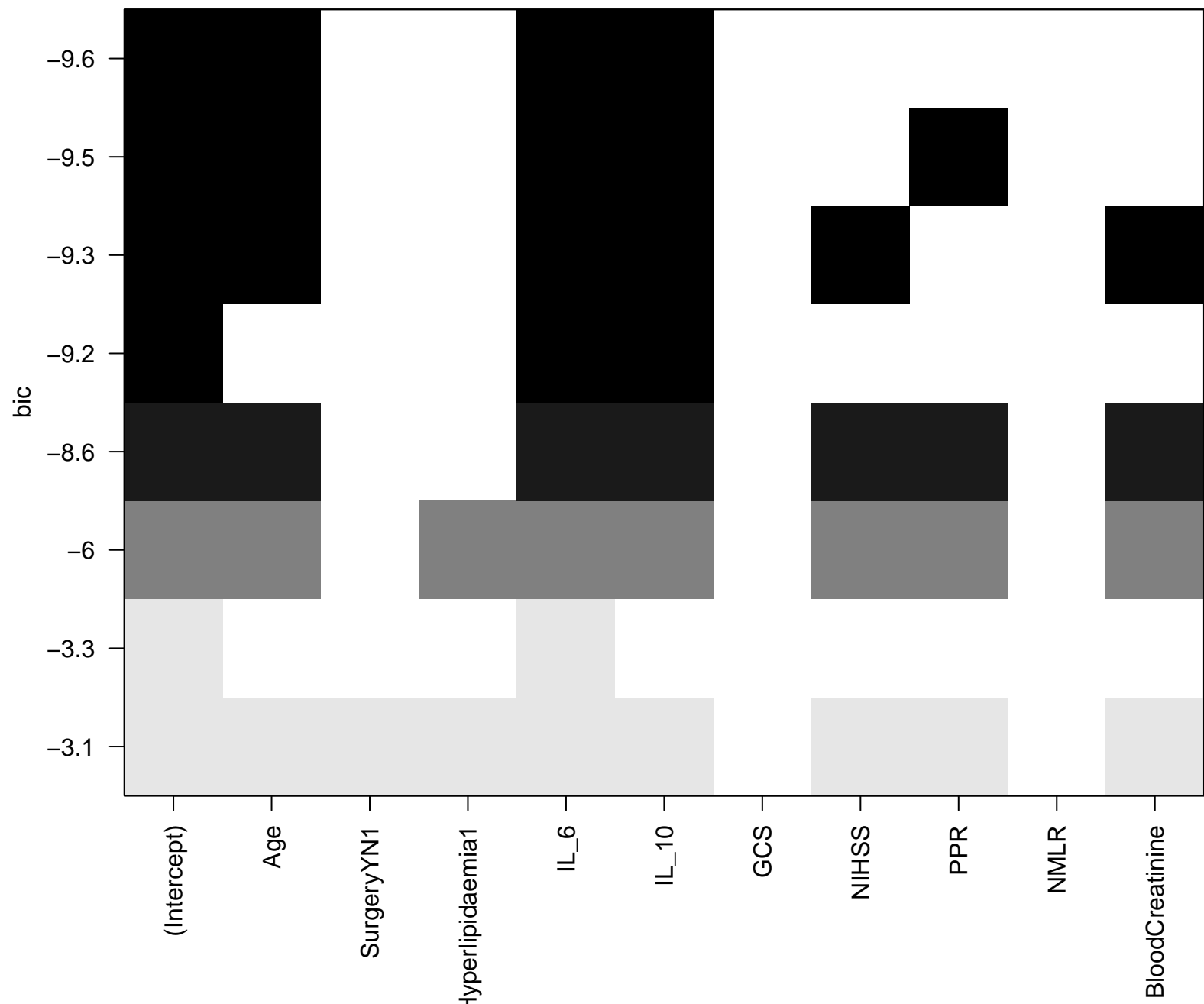

Supplement: Supplementary file 1 [file Data_Sheet_1.zip › 6.BIC.BSR.pdf]

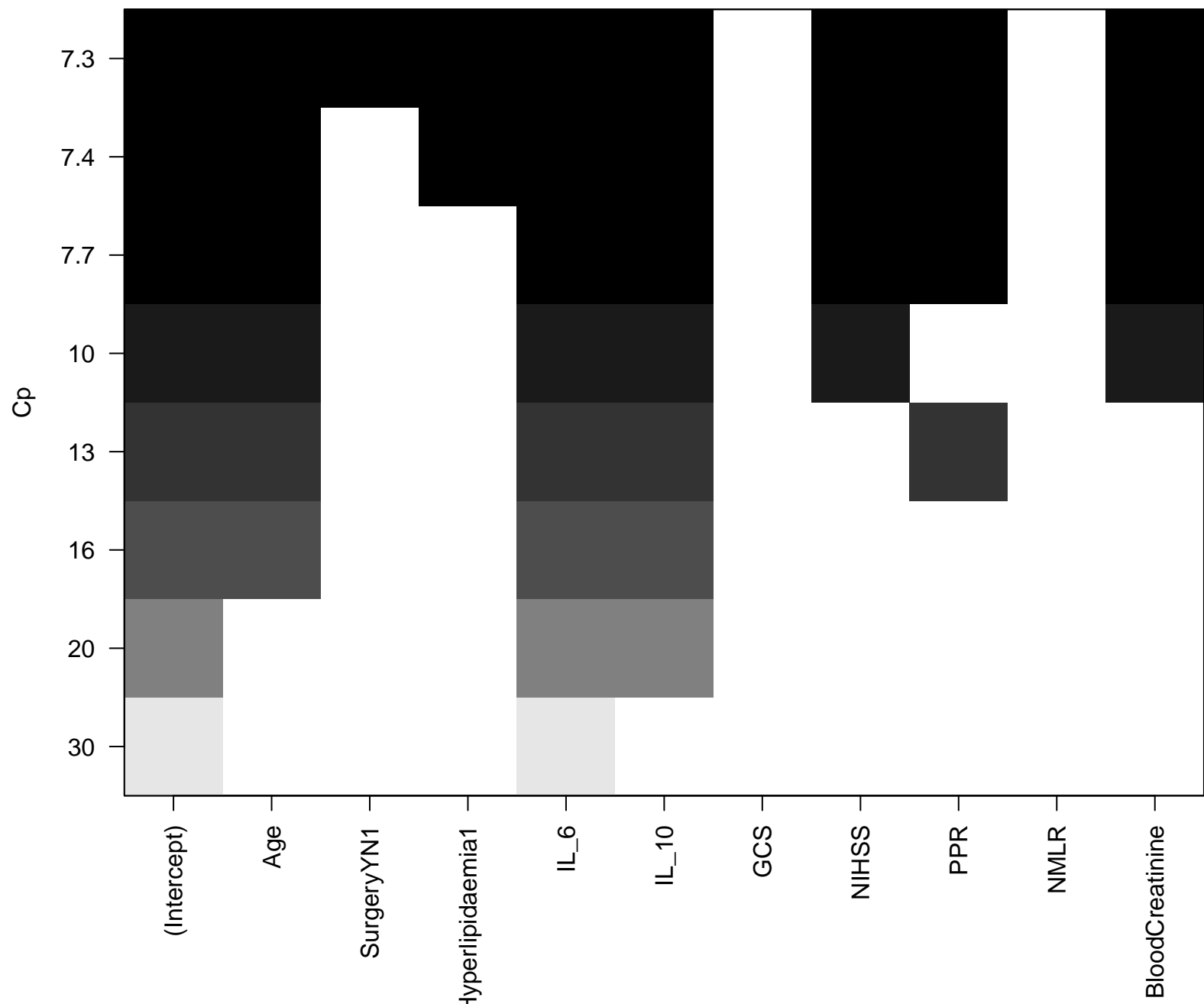

Supplement: Supplementary file 1 [file Data_Sheet_1.zip › 7.Mallows.Cp.BSR.pdf]

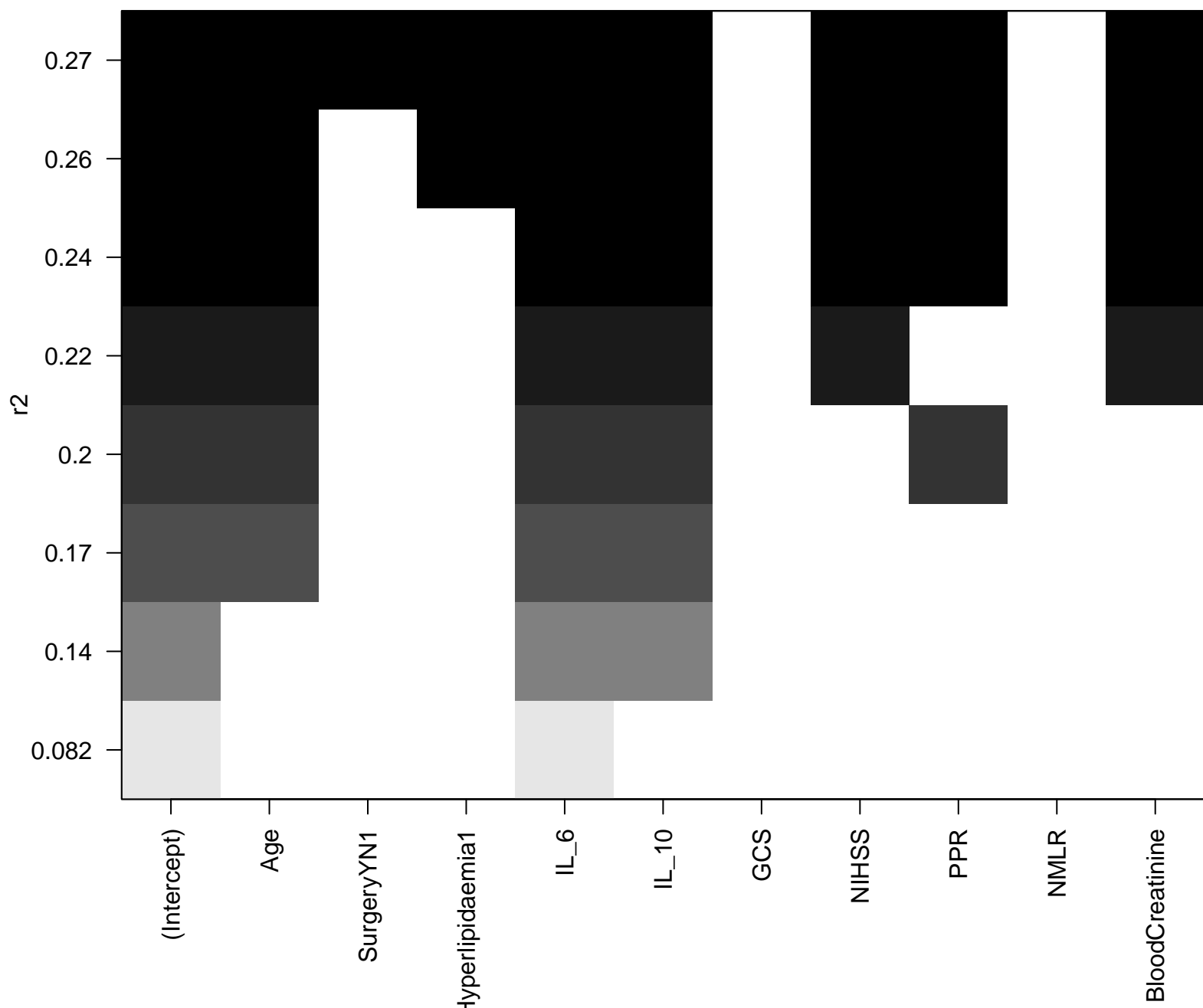

Supplement: Supplementary file 1 [file Data_Sheet_1.zip › 8.R-squared.BSR.pdf]

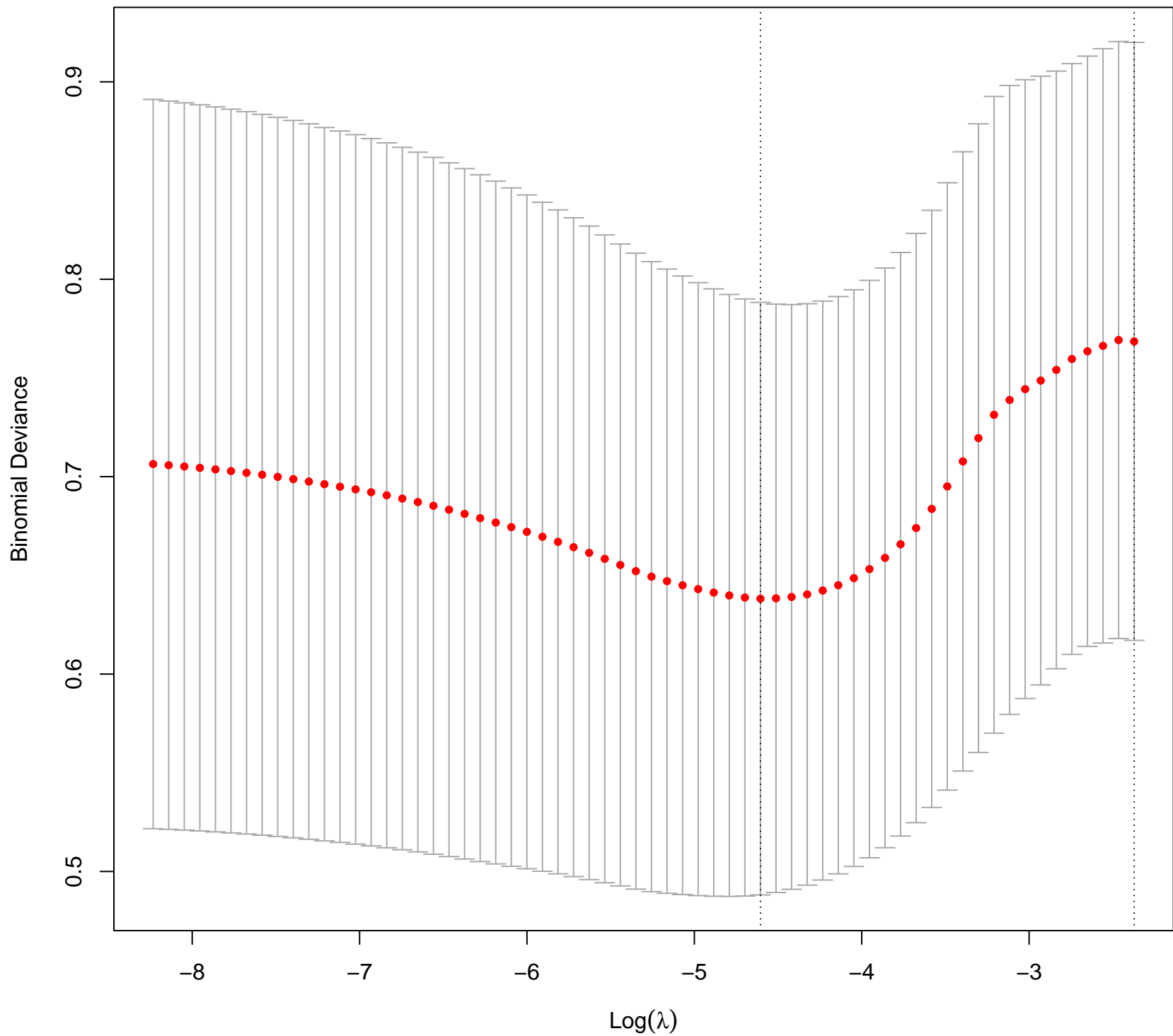

Supplement: Supplementary file 1 [file Data_Sheet_1.zip › 9.ridge.or.lasso.cross.validation.pdf]

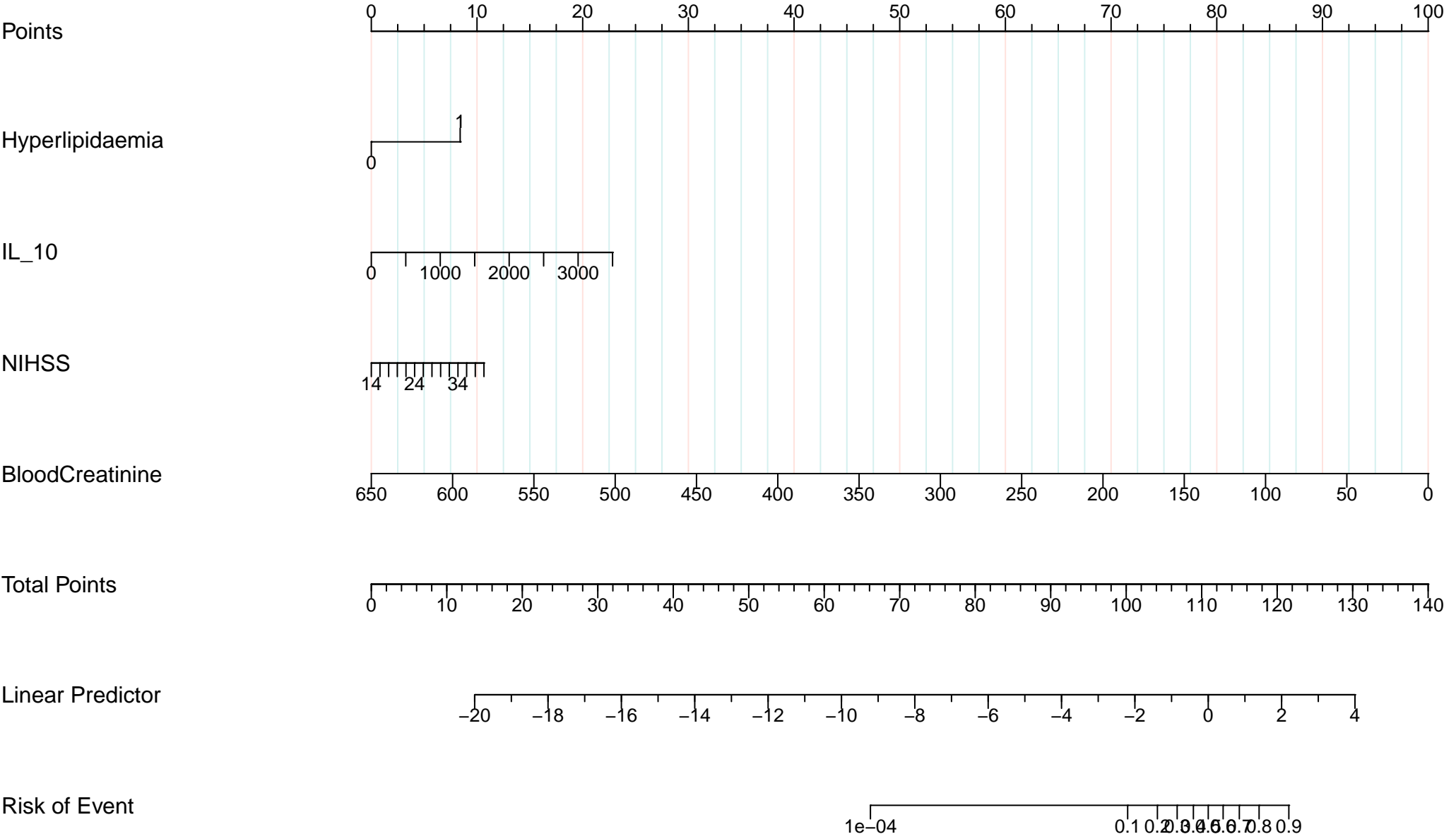

Supplement: Supplementary file 2 [file Data_Sheet_2.zip › 1.nomogram.pdf]

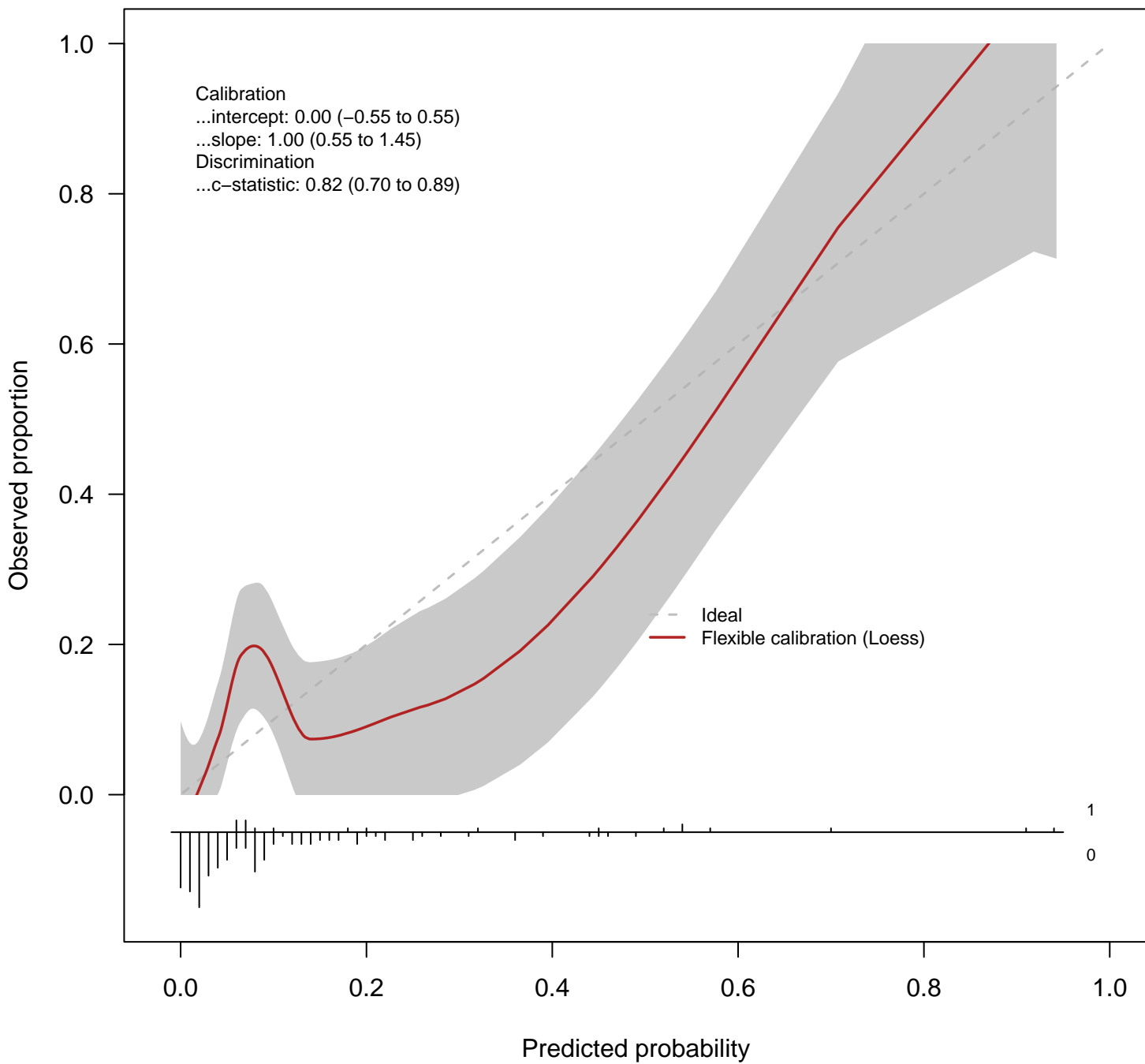

Supplement: Supplementary file 2 [file Data_Sheet_2.zip › 2.calibrate.LOESS.pdf]

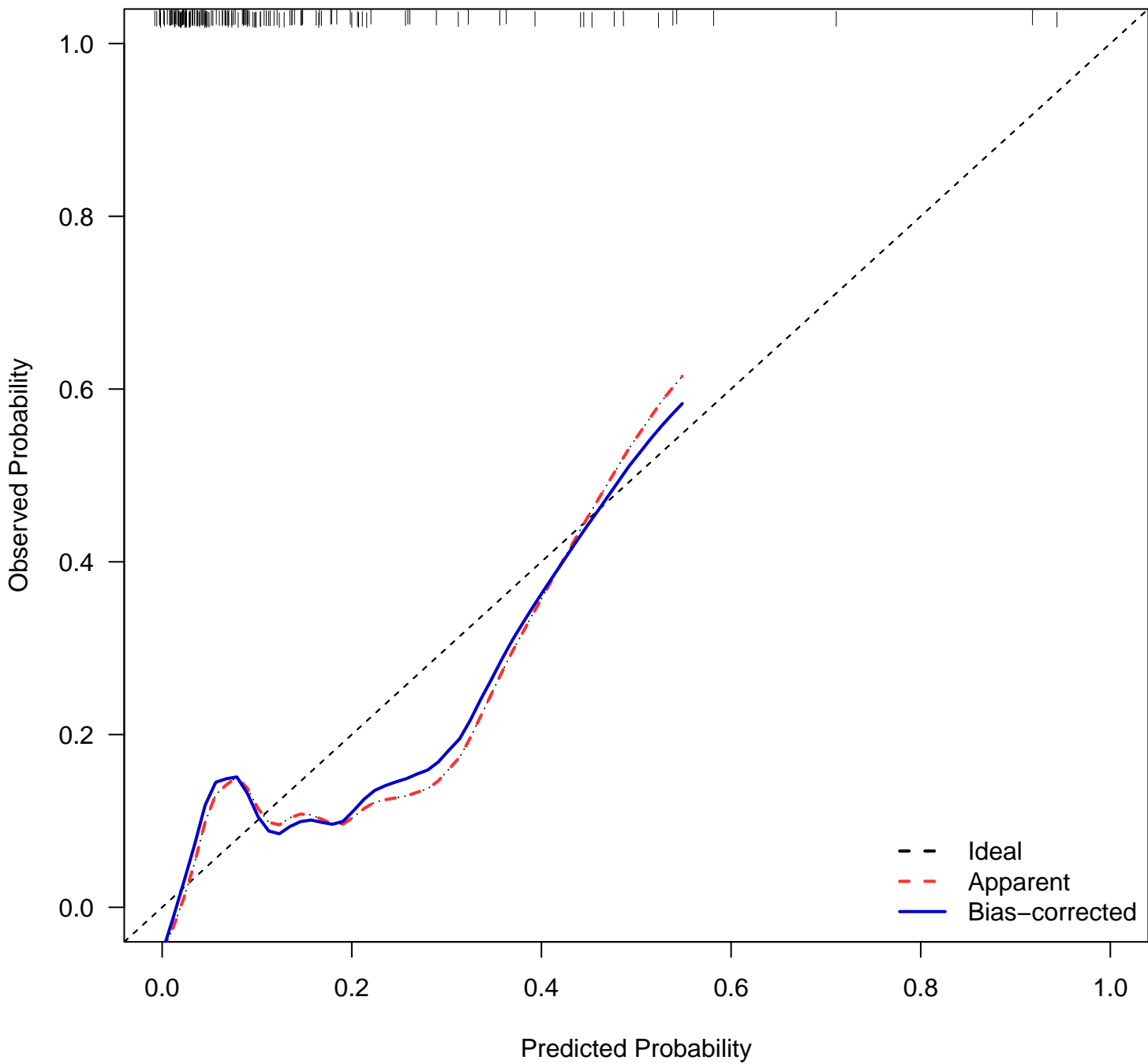

B= 200 repetitions, boot

Mean absolute error=0.047 n=157

Supplement: Supplementary file 2 [file Data_Sheet_2.zip › 2.calibrate.pdf]

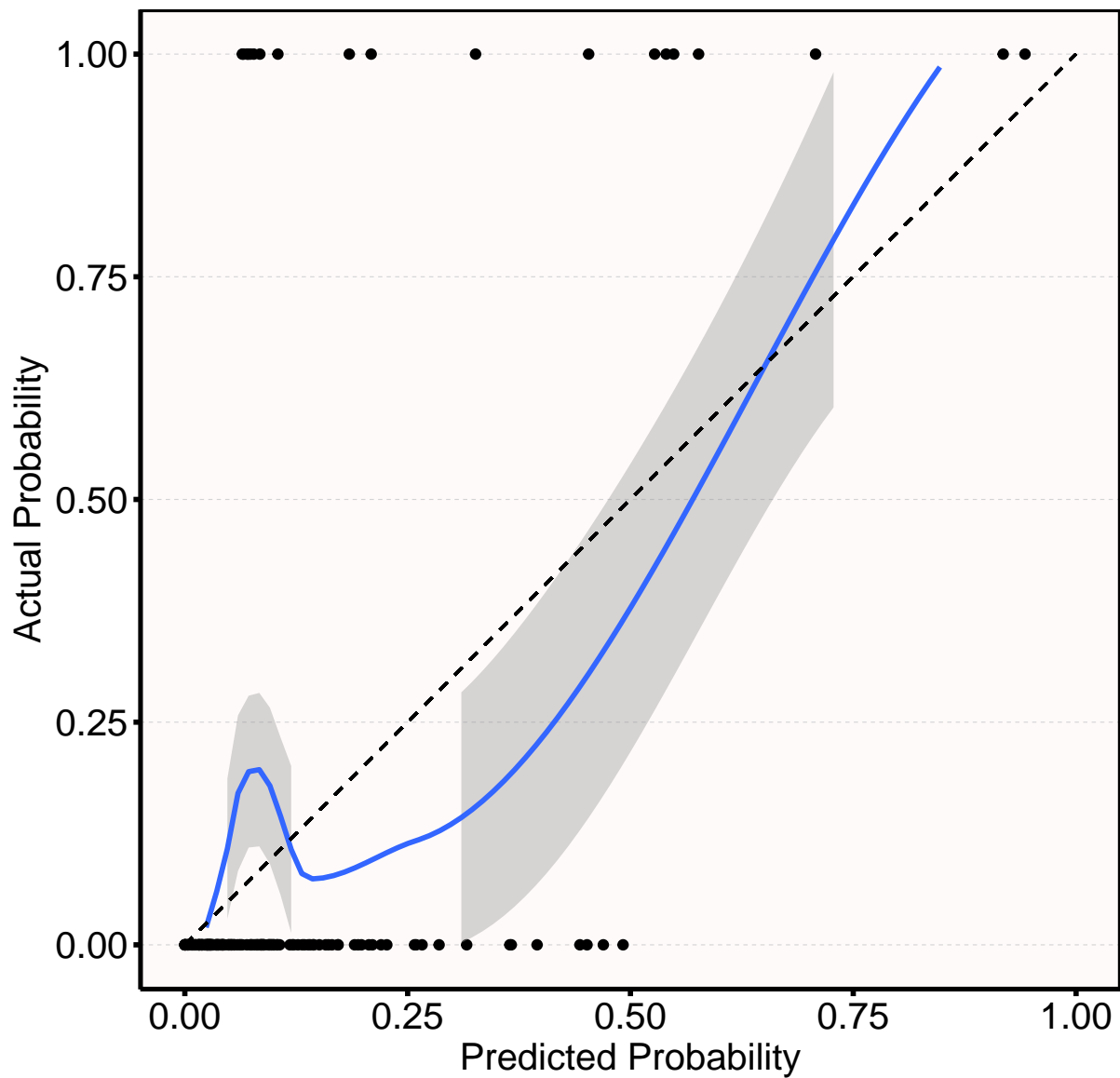

Supplement: Supplementary file 2 [file Data_Sheet_2.zip › 2.calibrate.smooth.pdf]

Standardized Net Benefit

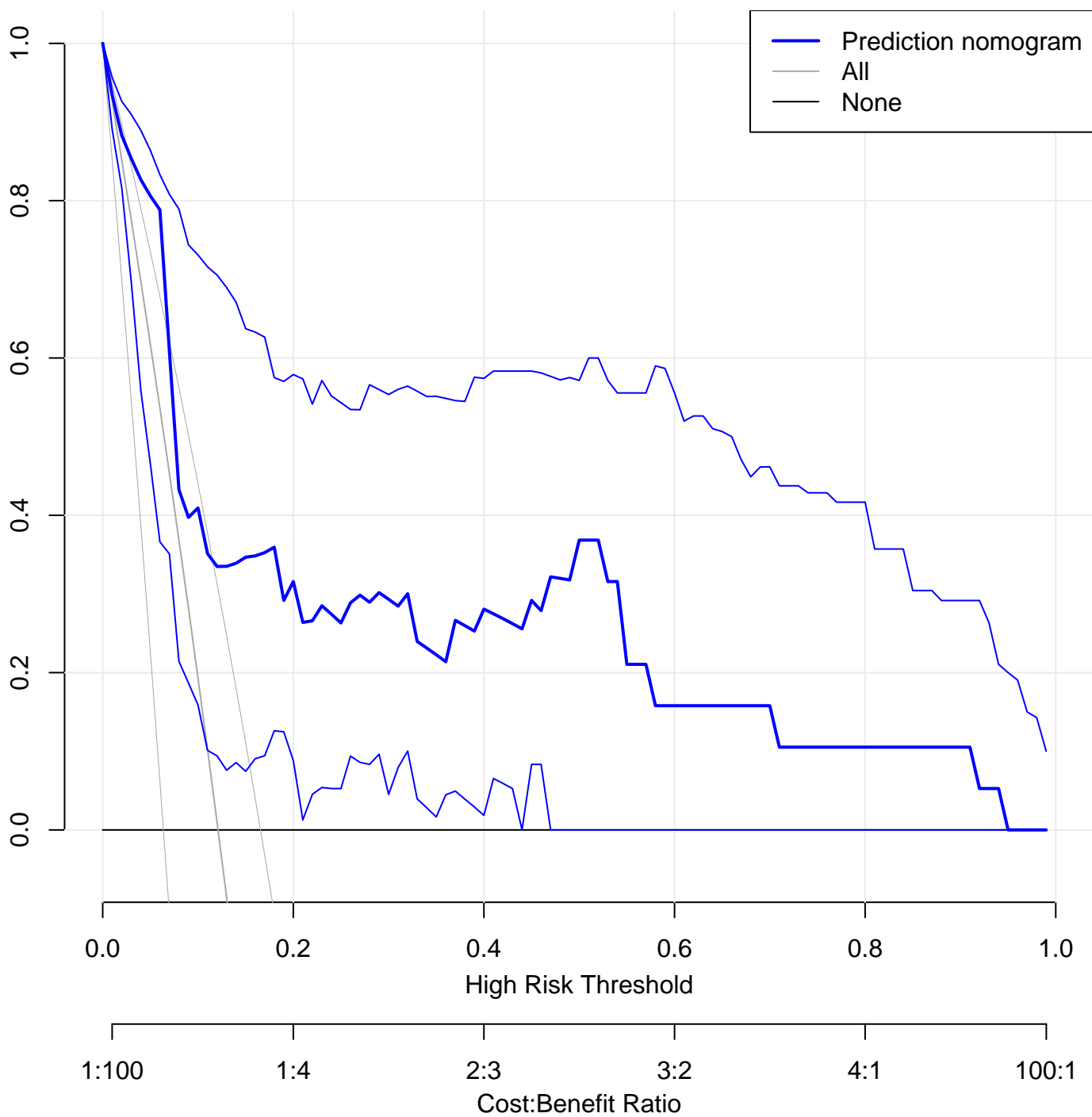

Supplement: Supplementary file 2 [file Data_Sheet_2.zip › 3.decision.curve.pdf]

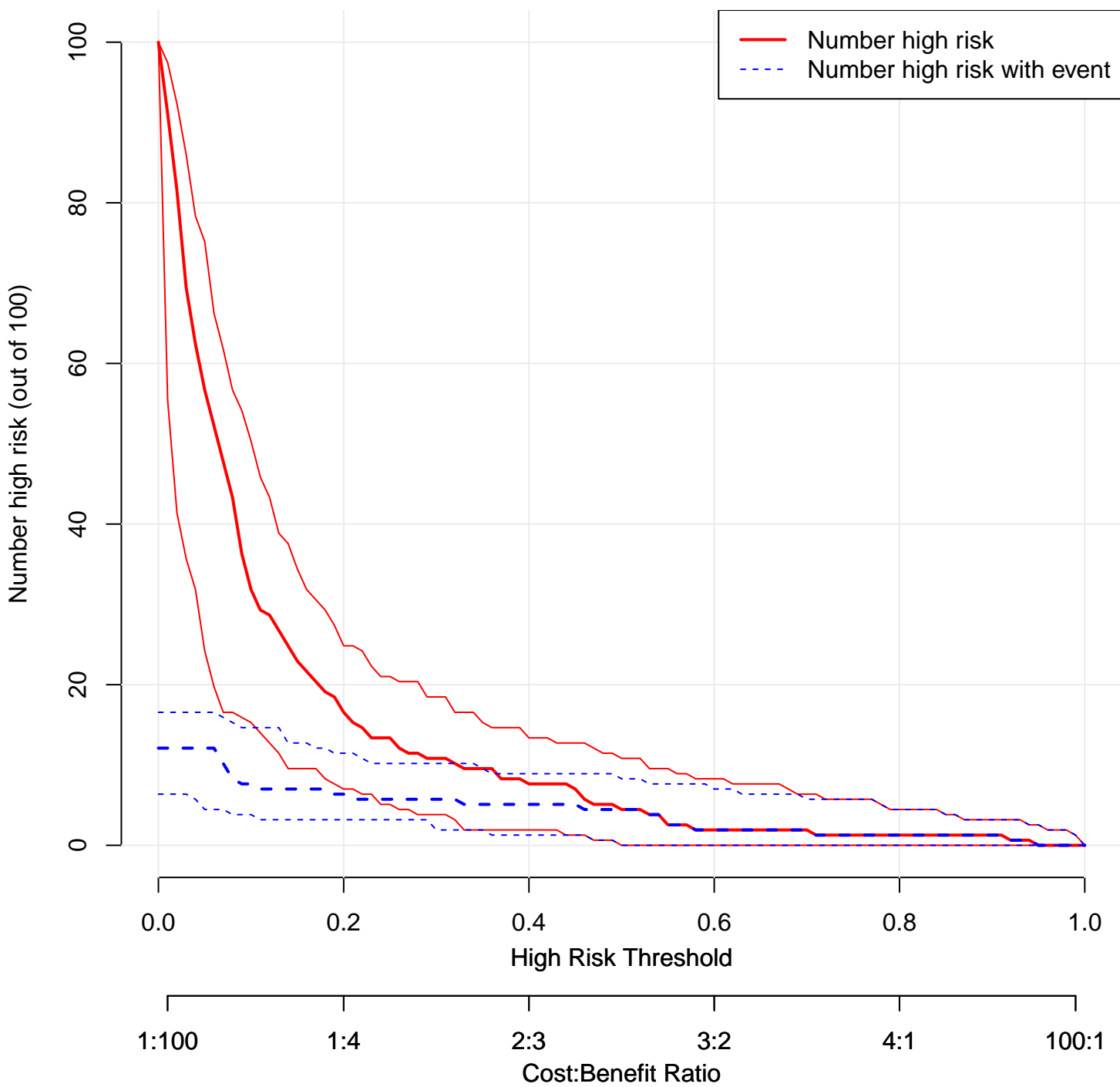

Supplement: Supplementary file 2 [file Data_Sheet_2.zip › 4.clinical.impact.pdf]

Model prediction

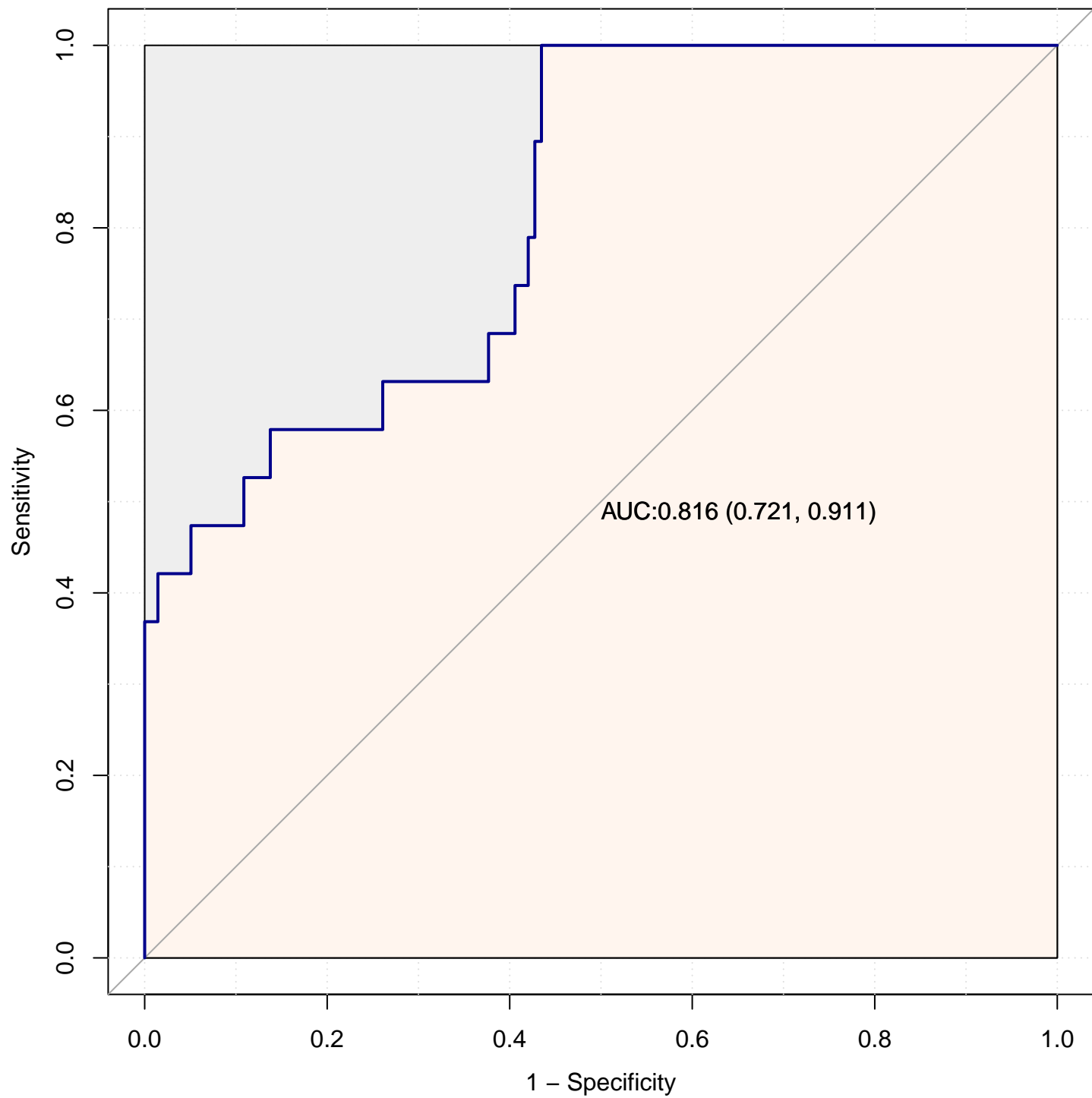

Supplement: Supplementary file 2 [file Data_Sheet_2.zip › 5.ROC.plot.pdf]

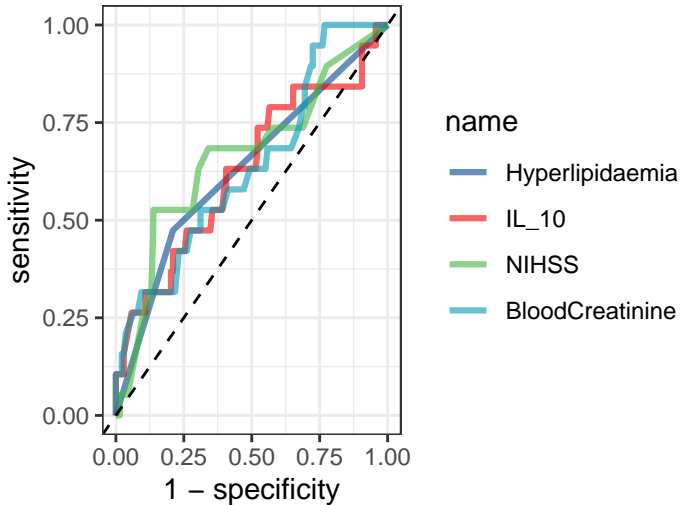

Supplement: Supplementary file 3 [file Data_Sheet_3.zip › ggROC.all.Plot.pdf]

model1\_prediction

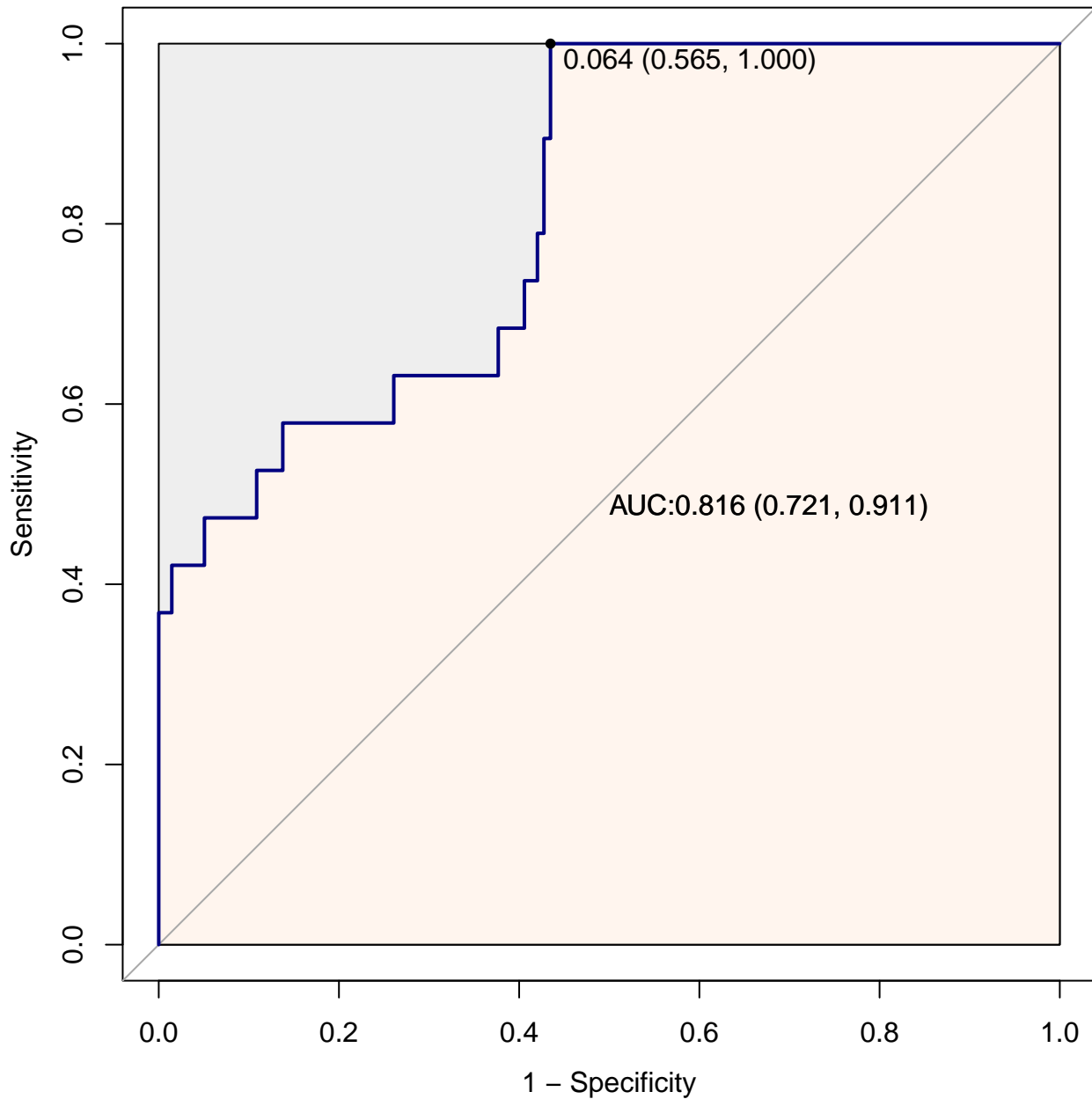

Supplement: Supplementary file 3 [file Data_Sheet_3.zip › ROC.plot.model1.pdf]

# Hyperlipidaemia

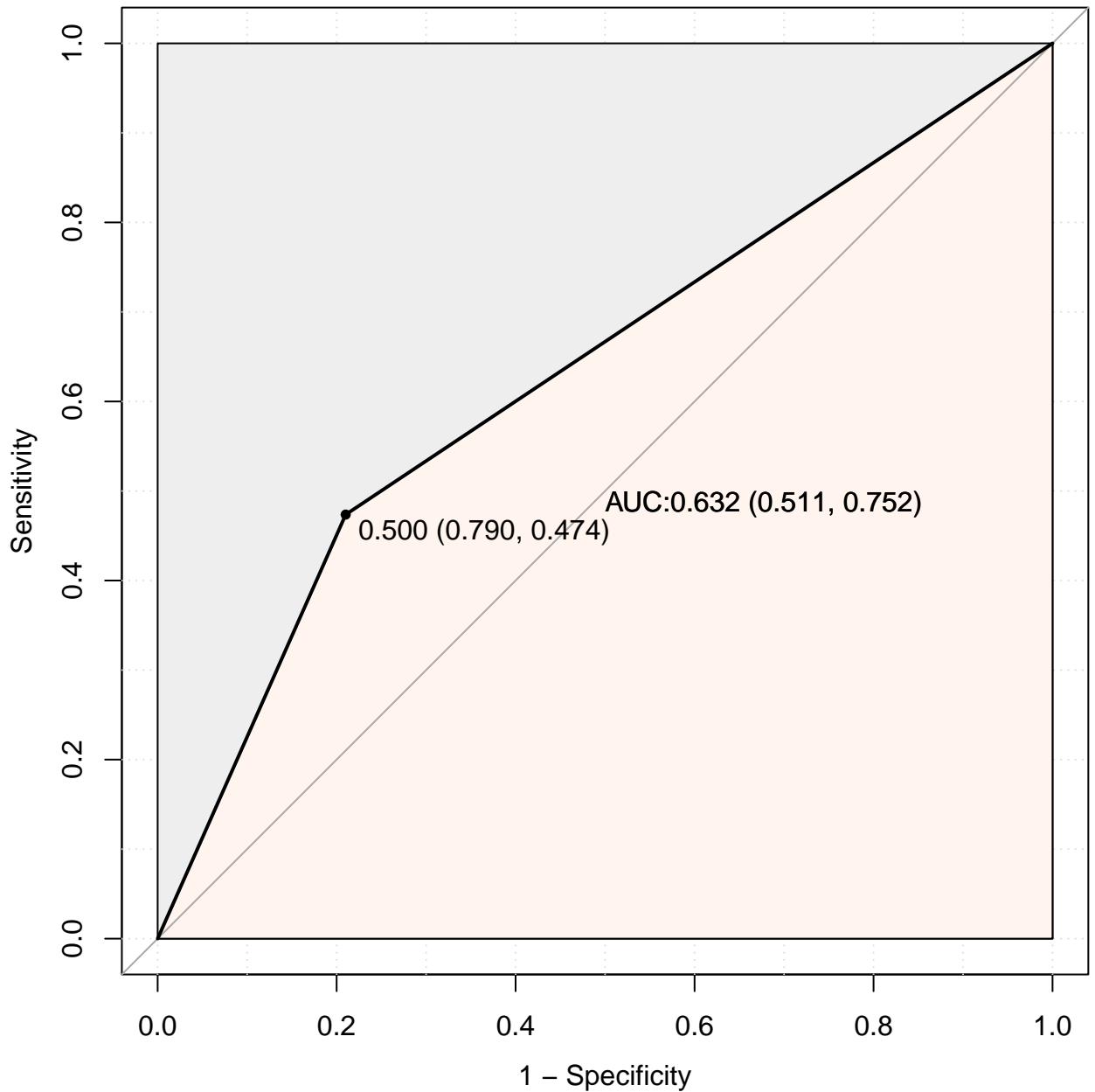

Supplement: Supplementary file 3 [file Data_Sheet_3.zip › ROC.plot1.pdf]

IL\_10

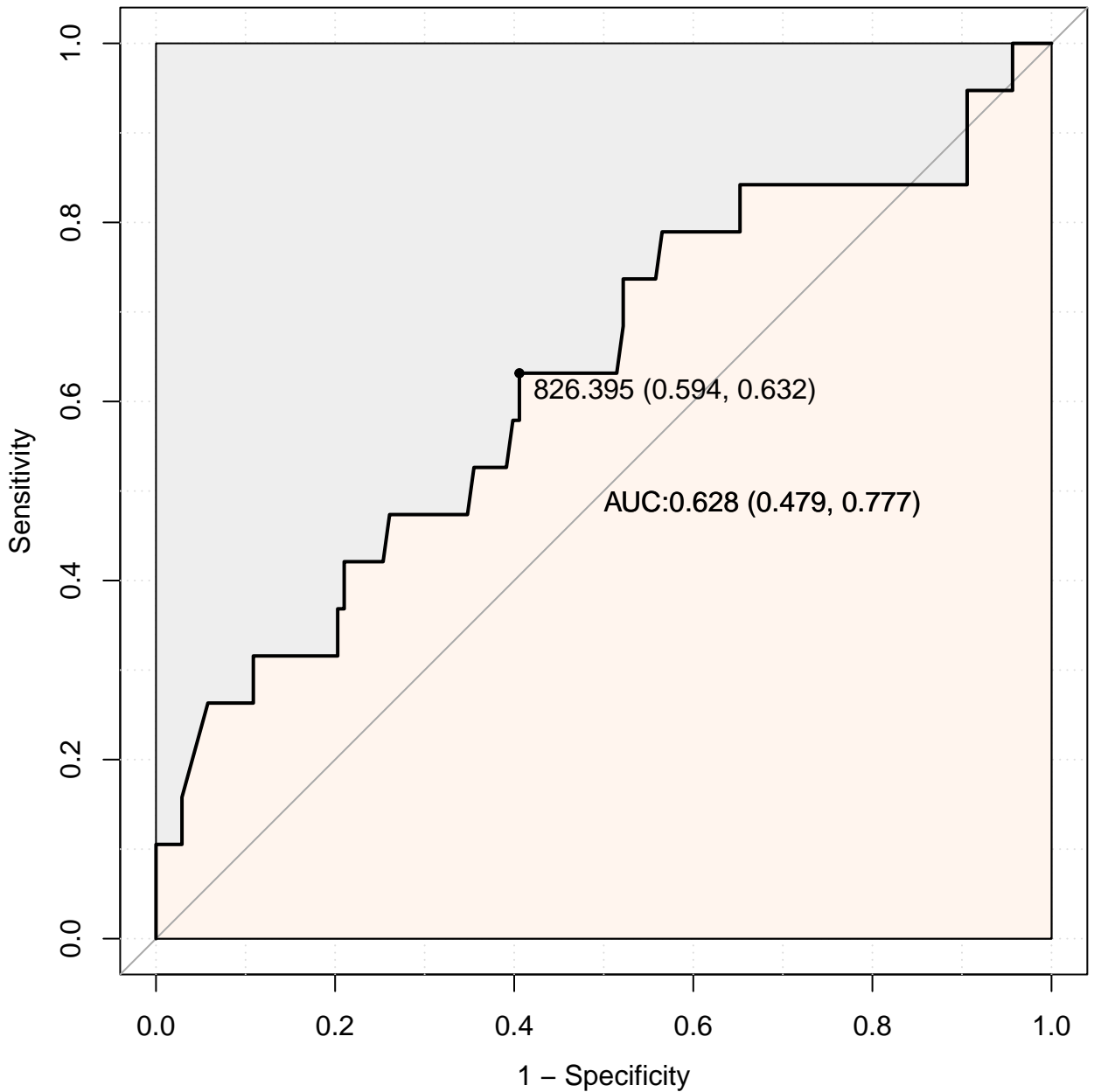

Supplement: Supplementary file 3 [file Data_Sheet_3.zip › ROC.plot2.pdf]

# NIHSS

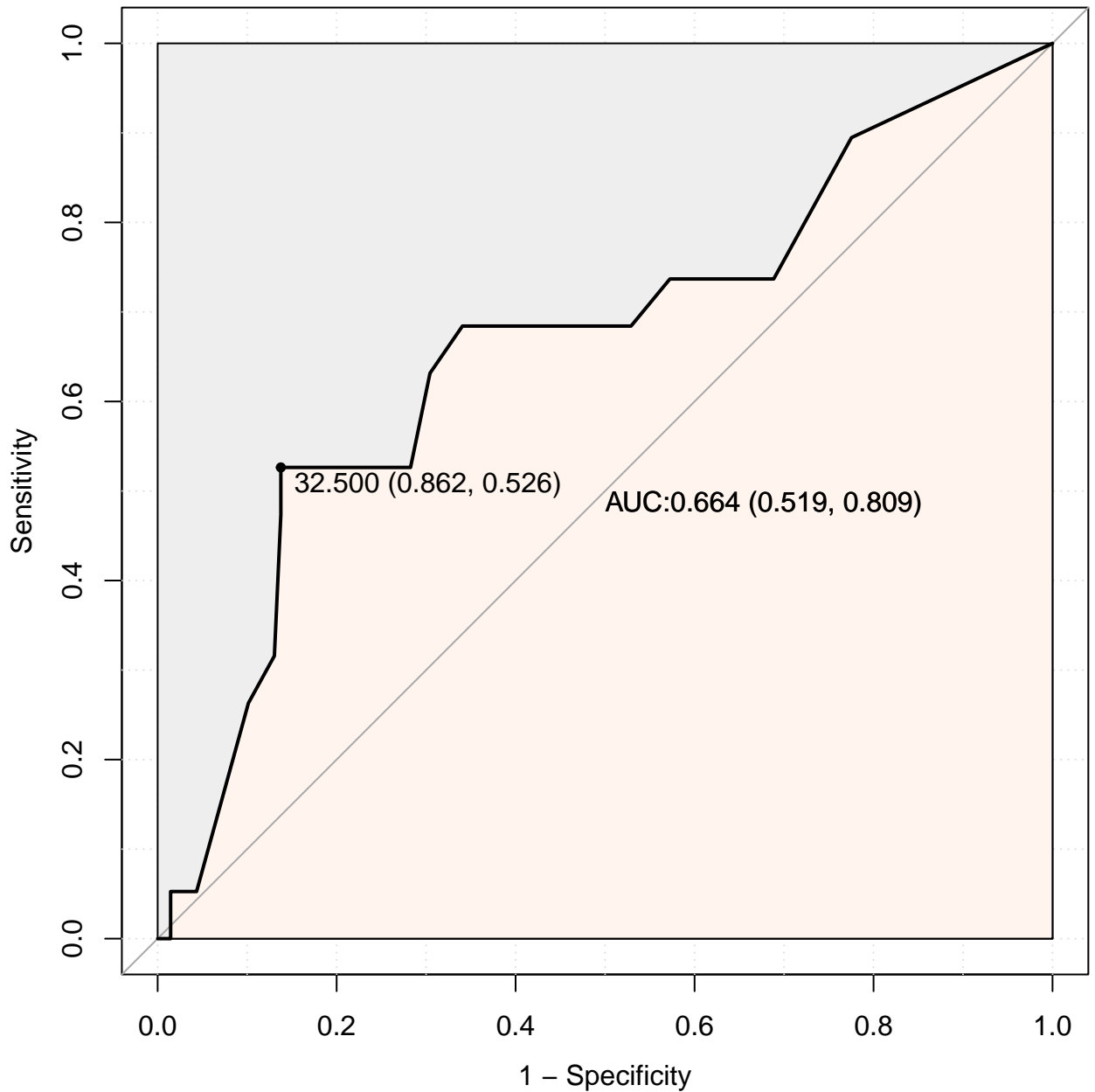

Supplement: Supplementary file 3 [file Data_Sheet_3.zip › ROC.plot3.pdf]

# BloodCreatinine

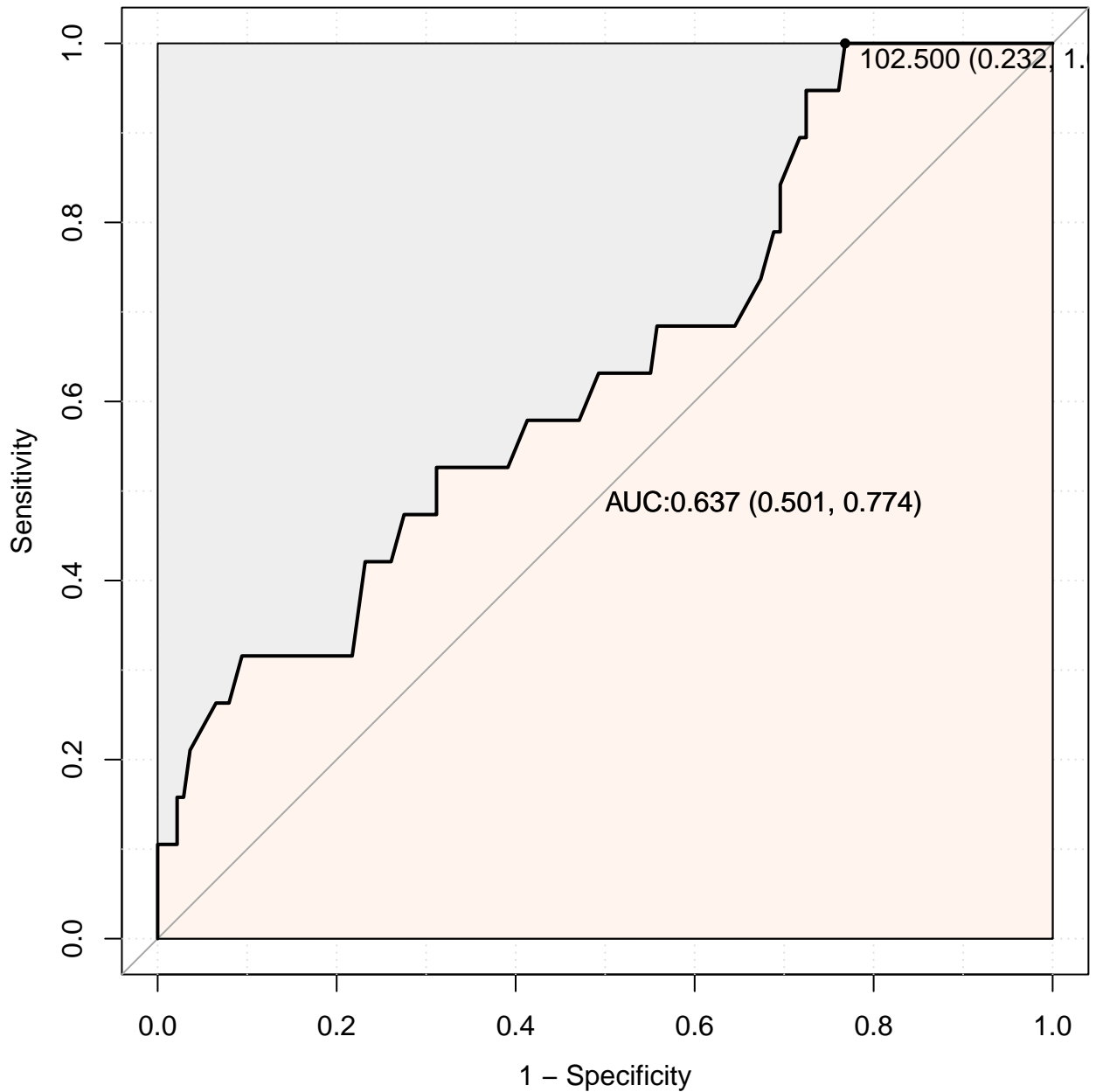

Supplement: Supplementary file 3 [file Data_Sheet_3.zip › ROC.plot4.pdf]
